# Genetic Characterization by SSR Markers of a Comprehensive Wine Grape Collection Conserved at Rancho de la Merced (Andalusia, Spain)

**DOI:** 10.3390/plants11081088

**Published:** 2022-04-16

**Authors:** Enrico Cretazzo, Paula Moreno Sanz, Silvia Lorenzi, Miguel Lara Benítez, Leonardo Velasco, Francesco Emanuelli

**Affiliations:** 1 Área de Mejora Vegetal y Biotecnología, Instituto de Investigación y Formación Agraria, Pesquera, Alimentaria y de la Producción Ecológica (IFAPA), Centro Rancho de la Merced, 11471 Jerez de la Frontera, Spain; miguel.lara@juntadeandalucia.es; 2 Dipartimento di Biologia Cellulare, Computazionale e Integrata, University of Trento, 38122 Trento, Italy; paula.morenosanz@unitn.it; 3 Research and Innovation Center, Fondazione Edmund Mach (FEM), 38010 San Michele All’Adige, Italy; silvia.lorenzi@fmach.it (S.L.); fra.emanuelli@gmail.com (F.E.); 4 Área de Protección Vegetal Sostenible, Instituto de Investigación y Formación Agraria, Pesquera, Alimentaria y de la Producción Ecológica (IFAPA), Centro de Málaga, 29140 Malaga, Spain; leonardo.velasco@juntadeandalucia.es

**Keywords:** microsatellites, grapevine germplasm, genetic diversity, eco-geographic differentiation, core collection

## Abstract

The IFAPA research center “Rancho de la Merced” (Jerez, Spain) hosts one of the oldest and most diverse grapevine germplasm repositories in Europe, and is aimed at providing feasible solutions to deal with any agronomic trait by exploring its genetic variability and by means of association and Deoxyribonucleic Acid (DNA) editing studies. In this work, we focused on a wine and dual-use grapevine subcollection that consists of 930 accessions. Genetic analysis allowed to identify 521 unique genotypes. After comparing them with several databases, matches were found for 476 genetic profiles while the remaining 45 have not been previously described. Combination with clustering analysis suggested a total pool of 481 *Vitis vinifera* accessions that included some table cultivars. Several synonymies, homonymies and mislabeling have also been detected. Structure analysis allowed identifying six clusters according to eco-geographic cultivation areas and one additional group including non-*vinifera* accessions. Diversity analysis pointed out that Spanish Mediterranean varieties are genetically closer to oriental genotypes than to European varieties typical of oceanic and continental climates. The origin of Spanish varieties is discussed in depth considering our data and previous studies. Analysis of molecular variance partition confirmed a well-structured germplasm, although differentiation among groups had a much lower effect on genetic variability than differences within groups, which are strongly related to a very high heterozygosity. A core collection that covers all allele richness is proposed. It is constituted of about 13% of total accessions, and each cluster inferred by structure analysis is represented.

## 1. Introduction

Germplasm repositories are a strategic resource for plant biodiversity conservation. Their efficient management and use are critical issues, especially in the case of field genebanks, of which the maintenance can be expensive. Well-managed plant collections both safeguard genetic diversity and make it available to breeders [1]. Grapevine (*Vitis* spp.) is a major vegetative propagated fruit crop with high socioeconomic importance worldwide [2], with a millenary history with lots of natural and human-mediated crossings and dispersions, originating thousands of varieties within the most economically important species in the genus, *Vitis vinifera* L. [3]. For these reasons, grapevine repositories have been established all around the world. The *Vitis* International Variety Catalogue (VIVC, https://www.vivc.de/index.php?r=cultivarname%2Findex (accessed on 12 April 2022) [4]) provides a comprehensive summary of worldwide grapevine collections and is making available a database with around 23,000 cultivars, breeding lines and *Vitis* species. For each, VIVC attempts to compile information about denominations (prime name and synonyms), historic references, passport data, morphological traits, use, molecular markers for identification, pedigree, etc. “Microsatellites by varieties” and “microsatellites by profiles” are especially useful sections, which support cultivar identification by providing genetic profiles of the nine Genres081/GrapeGen06 nuclear SSRs (Simple Sequence Repeat) markers VVS2, VVMD5, VVMD7, VVMD25, VVMD27, VVMD28, VVMD32, VrZAG62 and VrZAG79 [5]. Nuclear SSRs, due to their polymorphism, reproducibility, hypervariability and codominant nature, are an efficient tool to manage germplasm collections [6] and have been increasingly used as molecular descriptors in grape, which allow compilation, standardization and exchange of information concerning grapevine genetic resources [7]. The genetic structure, genetic diversity and cultivar parentage relationships have been inferred by SSRs in some of the widest grape genebanks, such as the ‘‘Domaine de Vassal’’ Grape Germplasm Repository at the “Institut National de la Recherche Agronomique” (INRA) (Montpellier, France, [8,9]) and the FEM grape germplasm collection at San Michele all’Adige (Trento, Italy, [10]). SSRs can also be successfully used, alone or in combination with other genetic or phenotypic markers, to extract subsamples known as core collections that capture, with minimum redundancy, most of the available genetic diversity of a crop, a wild species or a group of species, allowing a more effective management, research and utilization of the existing variation in a large collection [11]. Core collections have been inferred from grapevine repositories in order to manage a reasonable number of individuals to approach breeding programs [10,12,13,14]. Besides nuclear SSRs, chloroplast SSRs can help to disclose the geographic distribution of grapevine varieties and elucidate the relationships between *Vitis vinifera* subp. *sylvestris* and *Vitis vinifera* subp. *vinifera* grapevine groups [15]. In grapevine, chloroplast SSRs are maternally inherited and generate a small number of specific chlorotypes that can be related to a geographic origin. The VIVC recognizes this information for a consistent number of grapevine varieties.

In this paper, 930 *Vitis* accessions belonging to the “Rancho de la Merced” grape germplasm collection (IFAPA, Jerez de la Frontera, Andalusia, Spain) have been studied by 13 nuclear microsatellites. It is the most ancient and the second biggest grape genebank in Spain and one of the most important worldwide with approximately 1800 accessions among “*vinifera*” and “*sylvestris*” grapevines, *Vitis* species, rootstocks (RS) and interspecific crosses (IC). Its origin dates back to the foundation of the “Granja Escuela Práctica de Agricultura Regional de Jerez de la Frontera” in 1887, as a response to phylloxera appearance in southern Spain. In order to assist the local vitiviniculture facing this major concern, in 1907, the first Spanish ampelographic station was established in Jerez and included a collection of rootstocks and grapevine cultivars [16]. The germplasm collection was established at the “Rancho de la Merced” property in 1940, and between 1984 and 1987, was replanted and the number of accessions substantially increased. To date, only partial phenological and must quality characterizations are available [16]. A new replantation is now required due to the declined vineyard status. Therefore, the genotypic characterization by genetic markers is essential for a correct identification, which represents the first step in germplasm management [17].

Due to limited resources and practicality, replantation of the entire collection will not be done all at once, but by batches. The 930 studied accessions are planted in three plots assigned to the conservation of wine grape varieties for which replantation will be firstly achieved, since wine is largely the prevalent use for cultivated grapevine in both Spain and Andalusia (98% and 88%, respectively [18]). The rest of the repository is currently under genotypic identification. The 930 SSR profiles obtained have allowed detecting synonymies, mislabeling, redundancies and somatic mutants and will help to optimize the collection management. Genetic structure and diversity have been inferred, results have been integrated with information provided by the VIVC and compared with other related studies in order to discuss the origin of Spanish grapevine varieties. Provided that southern Spain viticulture is being especially concerned by main issues as climate change [19] and trunk diseases [20], two core collections based on SSR markers have been pointed out for further breeding programs.

## 2. Results

### 2.1. Genotypic Identification and Accession Denomination

According to registration data, the 930 accessions included in this study have been received from 33 locations belonging to 10 countries. In total, 496 (53.3%) come from Spanish donors, while 247 (26.6%) donors were Italian, French or Portuguese. The rest of the countries were only represented by few accessions (between 1 and 25). In seven cases, analysis of the two replicates per accession provided mismatching genotypes, so that the final number of analyzed accessions was 937 (Supplementary Material 1). Accessions from locations in Andalusia rise to 101 (73 were hold at an earlier “Merced” collection established in 1940 in the current germplasm location), while the donor is unknown for 153 (16.4%) accessions. For 891 accessions, a profile match was found in the databases consulted (mainly VIVC, see M&M). Table 1 shows a summary of the identification results. Wrong accession names were detected in 125 cases; in addition, 101 accession names did not correspond to any accepted denomination. Further verification should be performed to determine if they could be not yet recognized synonymies (Supplementary Material 1). In total, 4 of the 46 accessions with an unidentified profile did not have any given accession name and another 2 shared the same genotype, so that 45 profiles remained unknown. Altogether, there were 350 genotypes that were not duplicated, while the rest (587) were clones or sports of at least one other accession. The total number of unique genotypes was 521 and their classification according to information provided by VIVC is shown in Table 1.

Genotypes with an unknown utilization comprise the 45 unidentified profiles and cv. Gabriela. In total, 24 somatic mutants of 17 varieties and 2 from an interspecific cross were verified. In addition, four molecular variants (genotypes differing in just one allele) were detected for Colombard, Isabella (interspecific cross), and two accessions of Pinot Meunier. Finally, nine genotypes (including the two Pinot Meunier and four unidentified accessions) showed putative chimerism at least at one locus (see notes in Supplementary Material 1). The structure analysis, combined with Neighbor Joining (NJ) on total unique genotypes (Supplementary Material 5, Figure A), suggests that accession Churrín de Janeo (genotype 110, Supplementary Material 1), classified as *vinifera* in the VIVC, could be an IC or was used to obtain them. In fact, the two accessions whose profile matches with the putative *vinifera* Churrín de Janeo are denominated Híbrida and Híbrida Blanca, respectively (“híbrida” means hybrid in Spanish), and actually, their morphological appearance confirms the assignation to IC (personal observation). For Noah IC (genotype 110, Supplementary Material 1), information has been updated recently, and matches the genetic profile of accession Ondarrabi Zuri, but Cabello et al. [21] stated that a material identification mistake occurred in the “Finca el Encin” (Madrid) and that the accession present in this collection is indeed an IC, and not the true Ondarrabi Zuri, which is a *vinifera* cultivated in Northern Spain, which is known in France as Coubu. For the latter, all accessions grouping in sP7 (integrating admixed, see Supplementary Material 3) should be considered non-*vinifera*, except Khusaine Belyi, Dabouki and Morellone cvs and the accession named Patricia. Four IC, Gf.Ga.5242, Orion, Phoenix and Léon Millot, fits in sP6 (integrating admixed). We also excluded from the *vinifera* pool the accession Ikawa Opale (that groups in sP3) based on NJ and VIVC information.

### 2.2. Genetic Structure

**Figure 1:**
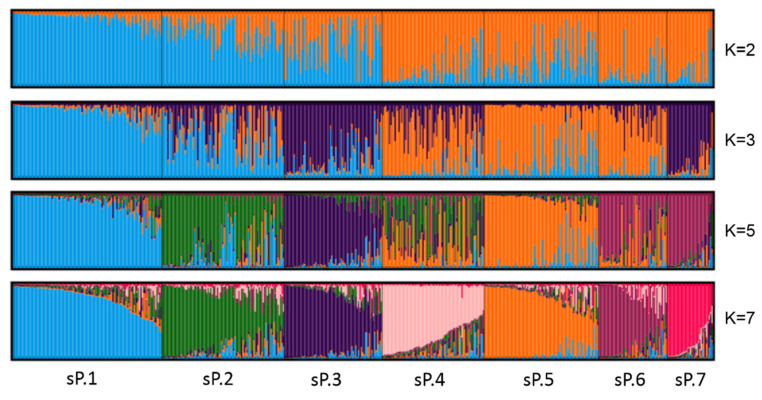
Inferred population structure of the collection using the model-based program STRUCTURE. K indicates the number of clusters (grouping level) set by STRUCTURE in order to investigate how individuals best split in different groups. sP means subpopulation. sP1: mostly central and southern Spain varieties, sP2: mostly varieties belonging to prole *pontica*, sP3: mostly varieties belonging to prole *orientalis*, sP4: mostly northern Italy and French varieties, sP5: mostly French and Portuguese varieties, sP6: mostly French and German varieties, sP7: mostly non *vinifera* accessions.

The analysis of the genetic structure with STRUCTURE identified four main grouping levels. The mean log-likelihood curve did not reach a plateau and the standard deviations did not increase drastically. However, from K = 7 the slope slightly decreased, and L(K) showed a tendency towards a plateau. The ΔK criterion indicated K = 2 and K = 7 as the most pertinent levels of population subdivision (Supplementary Material 2). Subsequent K were also explored (K = 3 and K = 5). Very few individuals could be strongly assigned (Q ≥ 0.78) when more than seven inferred groups were considered. Therefore, we assumed that the germplasm collection analyzed here can be divided into seven subpopulations (sP 1–7) that represent the most complex individual allocation (Figure 1).

Plots were generated with the DISTRUCT software based on the Q-matrix consensus permuted across 10 replications for K = 2 to K = 6 using the CLUMPP software. Each accession’s genome is represented by a single vertical line, which is partitioned into colored segments in proportion to the estimated membership in the two, three, five and seven subpopulations.

Data obtained by STRUCTURE were combined with information concerning the geographical origin of each variety provided in the VIVC. This info has been assumed as the best option despite some inconsistences detected (see discussion). Within individuals displaying ancestry values above the chosen threshold (Q ≥ 0.78), the sP1 comprises 73 genotypes, 44 of which are Spanish varieties mainly cultivated in the Central and Mediterranean regions, 8 varieties are Portuguese, typically cultivated in the South-Central regions, and only 4 are French (Supplementary Material 3). The sP2 consists of 37 genotypes, mainly central-eastern Mediterranean wine varieties, including 15 from Italy (mostly used in the southern regions), 13 from Balkans and 4 from Spain. The sP3 is predominantly composed of dual-use, including almost all table varieties of this collection, Muscat flavor and some Sultanina-related cultivars. The sP4 allocates Italian (mostly cultivated in the northern regions) and French varieties, some of them are typical of southern and southeastern regions (Provence, Languedoc-Roussillon and Rhone Valley) and some others have an uncertain origin. The sP5 highlights 24 varieties from Portugal, especially cultivated in the north-central regions, 14 from France, mainly originating in the western regions (Bordeaux, South West and Loire Valley), and 8 from Spain, mostly distributed in the northern regions. The sP6 includes varieties from Central Europe and from France, most of them typical of the east-central regions (Burgundy, Champagne and Alsace).

Finally, the sP7 contains almost all non-*vinifera* genotypes, the only three recognized *vinifera* varieties clustering here were admixed. The percentage of admixed accessions ranges from 36% at K = 2 to 45% at K = 7. In spite of the changes in the admixture levels, the comparison between different clustering steps showed that sP1 (mainly central-Mediterranean Iberian Peninsula varieties) together with sP2 (central-eastern Mediterranean wine varieties) clearly differentiate already at K = 2 from sP4, sP6 and sP7 (which comprise mostly central Europe and non-*vinifera* genotypes); however, sP1 presented a lower admixture than sP2. It is noteworthy that all 73 genotypes composing sP1 were already well discriminated from K = 2 through all the other K values explored till K = 7. Subpopulations sP3 (dual-use and table varieties) and sP5 (northern Iberian Peninsula and western France), which were mainly admixed for K = 2, got differentiated from K = 3. At K = 5, all subpopulations were discriminated except for sP4 (north Italy and southern France) and sP6 (Central Europe), which presented very high and moderate admixture levels, respectively (Figure 1). The Discriminant Analysis of Principal Components (DAPC) multivariate model, also performed with the unique genotypes, identified five groups as the best population subdivision. This clustering fitted fair well with the subpopulations obtained for K = 7 in STRUCTURE. In fact, excluding sP7 (which includes rootstocks and interbreeding crosses) and sP4 (which contained a high level of admixture at K = 5), for a level of stratification DAPC = 5, this new analysis was able to assign each cultivar to its respective subpopulation inferred with STRUCTURE at K = 7 with a fitness between 76% (sP5) and 100% (sP2) (Supplementary Material 4; Table 2). *V. vinifera* genotypes belonging to sP 1–6 were used to perform an NJ tree, which was consistent with STRUCTURE results (Figure B in Supplementary Material 5). An additional NJ tree was performed including the set of 101 cultivars with an eco-geographical origin inferred by Emanuelli et al. [10] according to Negrul [22] to Page 4/16 visualize the distribution of the genotypes among Negrul’s proles: *pontica*, *orientalis* and *occidentalis* (Figures C–H in Supplementary Material 5).

**Figure 2:**
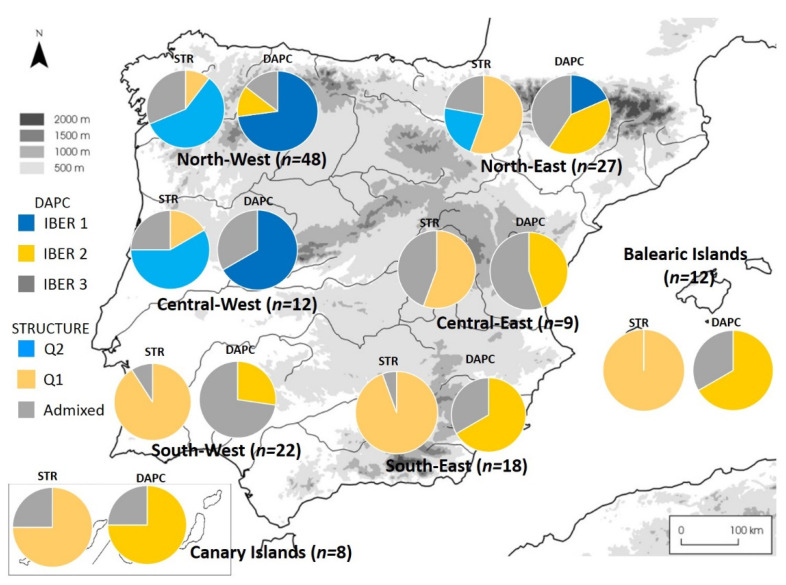
Geographical assignation of the Iberian Peninsula genotypes according to STRUCTURE and DAPC analyses. SRT and DAPC indicate STRUCTURE analysis and Discriminant Analysis of Principal Components, respectively. Q and IBER indicates the groups disclosed by STRUCTURE and DAPC analyses, respectively, on Spanish and Portuguese (Iberian) varieties. Admixed indicate individuals not clearly assigned to any group (the highest Q is ≤ 0.78).

An additional geographical assignation of Portuguese and Spanish genotypes (Supplementary Material 3; see M&M) allowed performing STRUCTURE and DAPC analyses of the Iberian Peninsula germplasm, disclosing two and three genetic backgrounds, respectively. In both cases, subpopulations were differentially distributed according to the area of origin/cultivation (Figure 2; Supplementary Material 6). In fact, one subpopulation was exclusive of the central-west and northern area, but with an opposite representation pattern between the north-west (NW) and north-east (NE). An opposite distribution pattern was also observed between the south-west (SW) and south-east (SE) areas.

Taking into account the analysis performed with STRUCTURE on the whole dataset, genotypes within IBER 1/Q2 grouped mainly in sP5 (northern Iberian Peninsula and western France), while those within IBER 2 and IBER 3/Q1 grouped for the most part in sP1 (central-Mediterranean Iberian Peninsula varieties).

STRUCTURE analyses best fit at K = 2, while DAPC analyses disclose three main subpopulations.

### 2.3. Genetic Diversity and Genetic Differentiation

**Figure 3:**
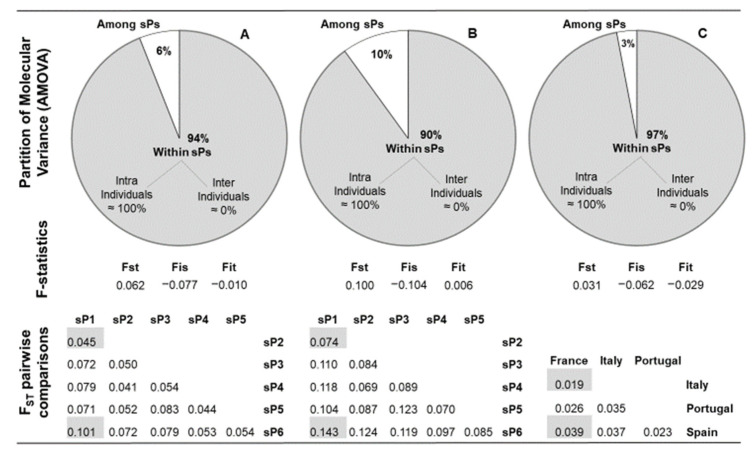
Hierarchical genetic variation and genetic differentiation among and within groups of varieties/accessions. sP means subpopulation (see Figure 1). (**A**): subpopulations inferred by STRUCTURE according to the highest Q (membership coefficient); (**B**): subpopulations inferred by STRUCTURE according to Q ≥ 0.78, (**C**) Most represented countries (variety origin from VIVC). In order to perform F-statistics via AMOVA, a Codominant-Allelic genetic distance matrix was generated. i, s and t indicate individual, subpopulation and total, respectively. Absence of population differentiation (F_ST_ ≈ 0) was assumed as null hypothesis (H0), with F_ST_ > 0 as an alternative hypothesis (H1), and observed values of F_ST_ were compared with 1000 permutated values. All F_ST_ values in pairwise comparisons are significant (*p* ≤ 0.001).

These analyses were performed using the pool of 481. *V. vinifera* genotypes. The PIC for each locus is shown in Table 3. The most polymorphic marker resulted to be MD28, while the least was ISV3. The second lowest value was for MD25, which showed only five alleles with a frequency of more than 1%. Four markers (ZAG79, EVA2, VVS2 and MD28) showed the highest number of alleles (11) with a frequency of more than 1%. ISV2 and ZAG79 presented, respectively, the most and the least number of rare alleles. Genetic diversity parameters are shown in Table 4. Cluster values of He ranged from 0.695 to 0.809 with a clear increase when groups included admixed genotypes. Ho was always higher than He, which means a slight excess of heterozygosity (F < 0). The PI is around 10–17, suggesting that identical genotypes with different denominations should correspond to synonyms. All AMOVAs (Analysis of Molecular Variance) performed with distinct pools (Figure 3A–C) show a narrow differentiation among groups, with the total variance being mostly dependent on differences within clusters. The F statistics confirm the excess of heterozygosity (FIS < 0), remarking that individual loss of heterozygosity versus total population does not occur in any case. The Mediterranean Iberian subpopulation (sP1) shows the closest genetic relationship with sP2, while the farthest is with sP6, given that sP7 has been excluded because it is mainly composed of non-vinifera individuals.

### 2.4. Core Collectionsgenetic Diversity and Genetic Differentiation

Based on the M-method, 35 cultivars (core-35) were sufficient to capture all the 112 alleles occurring with a frequency more than 1%. The core-35 was then used to design the final genetic core collection retaining 100% of SSR diversity, i.e., 168 alleles. The optimal size of this core was 63 individuals (core-63); thus, 28 accessions were added at this step to retain 56 rare alleles (Supplementary Material 3). In both collections, members from each inferred cluster by STRUCTURE at k = 7 are included, with the sP1 being the most represented and sP6 and sP7 the least ones, as could be expected according to different sP sizes (Table 5). Strikingly, sP2 is underrepresented in core-35 with respect to core-63, in which percentages between groups containing a major number of accessions (sP1, 2, 3, 4 and 5) are more similar. Heterozygosity values are in the same range of the full collection and inferred clusters.

## 3. Discussion

### 3.1. Genotypic Identification and Accession Denominations

Establishing the geographical origin and the correct prime name of a grapevine variety can become difficult due to the existence of a great number of synonyms and homonyms locally used, especially in the Mediterranean basin, as a result of the human displacements and migration through the centuries [3]. Therefore, population structure analyses such as those performed with STRUCTURE or Darwin software could be helpful to solve doubts about the correct geographical assignation. A consistent number of mislabeling and not verified denominations have been detected, as well as some discrepancies in allele size with respect to VIVC and other databases (Table 1 and Supplementary Material 1). Despite VIVC being continuously updated, the volume of data managed is very large and possibly still contains some not fully revised information collected before the GrapeGen06 SSR set, which was provided as a common SSR coding method. Some VIVC prime names of Spanish varieties do not correspond to the prime names reported in the commercial variety national catalogue (https://www.mapa.gob.es/app/regVar/ResBusVariedades.aspx?id=es&TxtEspecie=VID&IDEspecie=119 (accessed on 12 April 2022)) (e.g., Albillo Forastero instead of Forastera Blanca or Mouratón instead of Juan García). A list of changes will be privately suggested to JKI; in this work, Blanca Gordal has been assigned for genotype 63 (Supplementary Material 1) instead of Corazón de Cabrito (VIVC variety number 24550), since it is the denomination recently recognized by the Spanish Office of Vegetable Variety (OEVV, https://www.mapa.gob.es/app/regVar/DetalleVariedad.aspx?id=es&TipoV=C&IDVariedad=20160147 (accessed on 12 April 2022)).

For varieties Cañocazo and Malvasia di Lipari (genotypes 90 and 243, Supplementary Material 1), we could not find a clear correspondence with any VIVC variety number, although these materials have been long managed by us with a degree of certainty concerning varietal identity. Further checks will be performed as soon as possible to clarify this situation.

For a number of unidentified genotypes, correspondences have been found recently, after data analyses, and information has been updated in Supplementary Materials 1 and 3 during paper revision.

Regarding the twenty-four somatic mutants found, nineteen are berry color sports, two are pulp pigmentation mutants (Gamay Teinturier de Bouze and G.T. Freaux) and three are leaf morphology sports (Supplementary Material 1).

Finally, it is worth noting that Regent, Sirius, Phoenix and Staufer are commonly considered as *vinifera* varieties; however, based on strict botanical criteria, they are interbreeding crosses (see Supplementary Material 1). To date, there is no international requisite to establish which percentage of *vinifera* genome is enough to assume an accession as a *V. vinifera* variety.

### 3.2. Population Structure, Genetic Diversity and Genetic Differentiation

Grapevine collections are germplasm repositories built over several decades through different networks of national and international partnerships. However, all of them are far from comprising all grapevine cultivars worldwide, of which approximately 10,000 are estimated to be held in field collections, in addition to an undefined number of local minority grapes not yet prospected [23]. Therefore, although several studies have explored the genetic information of these germplasm repositories, none could be fully conclusive about the genetic structure of the entire cultivated grapevine gene pool; the most comprehensive approach was based on 2096 cultivars from 52 countries [9]. The collection studied here is almost entirely composed of wine and dual-use varieties and about 75% of the accessions supposedly originated in Central and Western Europe. In any case, the genotype partitioning in the STRUCTURE subgroups seems to be stable even when the dataset analyzed presents an unbalanced repartition of genotypes from the different regional groups [9]. The *vinifera* pool of the grapevine collection characterized in this study showed a great He (Table 3), similar to that displayed in larger collections [9,10]. In such high genetic variation conditions, only minimal gains in terms of total variability are possible through extending the genetic pool with entries from diverse eco-geographic sources [24]. Therefore, genetic structure and diversity studies may assist association studies.

The ΔK criterion give rise to the first structural level in the data [25] that depends on the nature of the samples analyzed. In the present study, the highest value was obtained for K = 2 (Supplementary Material 2) that split up accessions cultivated in the Mediterranean climate from those of the Oceanic and Continental climates (Figure 1), unlike previous reports where SSRs at K = 2 distinguished among *V. vinifera* and non-*vinifera* [10], between subsp. *vinifera* and *sylvestris* [26], or among proles and specific subproles [13] according to Negrul’s classification [22]. At K = 3 an additional cluster containing non-*vinifera* genotypes as RS and IC, table grapes and others with dual-use was pointed out. At K = 5 and K = 7, the grouping proposed by Negrul [22] was retraced with some additional partition. Varieties in sP2 (central-eastern Mediterranean wine varieties) and sP3 (dual-use and table varieties) essentially belong to proles *pontica* and *orientalis*, respectively, and this is consistent with Darwin trees obtained combining our data with eco-geographic groups inferred at FEM [10] (Figures C–H in Supplementary Material 5). Interestingly, Tempranillo, the most cultivated variety in Spain, and Tinto Velasco, also very interesting in Andalusia for its flavor and drought adaptation, fit in sP2. According to Terral et al. [27], Tempranillo shows morphological similarities with some ancient French varieties; in our opinion, this could be related to the not fully disclosed origin of both Tempranillo parents, Albillo Mayor and Benedicto [28]. The origin of Tinto Velasco is still under investigation [21]. The sP4 is primarily composed of northern Italy varieties and of some from south-eastern France, which are mainly admixed at K = 5. In particular, Italian genotypes presented a very high admixture percentage (Supplementary Material 3) in each level according to the weak structuration detected by Cipriani et al. [29]. French varieties also present a high admixture level at K = 5 and K = 7.

It is worth noting that they mostly split into sP4, 5 and 6 according to regional cultivation areas, similarly to Aradhya et al. [24]. Instead, Spanish varieties show very low admixture levels, especially at K = 2 and 3, in disagreement with Bacilieri et al. [9] and Laucou et al. [30], although this probably depends on the nature and composition of the set of samples analyzed. Both Spanish and Portuguese cvs. mainly split into sP1 (Mediterranean Iberian Peninsula, mainly proles *orientalis* in NJ, Figure C in Supplementary Material 5) and sP5 (northern Iberian Peninsula and western France, proles *occidentalis* Figure G in Supplementary Material 5), but the relative proportion is the opposite, probably because in Spain as a whole the Mediterranean climate prevails, whereas in Portugal the Oceanic one prevails. The constitution of sP1, which includes most of the Spanish varieties from the Mediterranean climate, some Portuguese and a few French ones, fits with the cluster identified in the largest grapevine collection worldwide by the 18 k SNP (Single Nucleotide Polymorphism) genotyping array [30].

All *V. vinifera* groups inferred by STRUCTURE showed consistent genetic diversity (He) in the same range of previous reports [9,24,26]. In all cases, the value increased only by less than 1% when the most permissive level of ancestry is considered (Table 4). Each group presented a slight excess of heterozygosity with respect to Hardy–Weinberg equilibrium that is supported by the negative values of F. Only sP6 He is slightly below 0.7 probably due to the low number of individuals with strong ancestry. The sP3 should be expected to have the biggest value because of the higher diversity contained in the proles *orientalis* [9], but probably, the number of individuals with Asiatic origin included in this collection is not enough to confirm this hypothesis. Portuguese cultivars presented the highest diversity among the main represented countries (Table 4). F_ST_ values were statistically consistent. Nevertheless, only the general tendency can be compared with other studies because of the different definitions, estimation methods and interpretations of F_ST_ generate some confusion in the literature [31].

Mediterranean Spanish cvs. (sP1, mainly allocated within the proles *orientalis*-*antasiatica* in NJ, although some accessions laid within the proles *pontica*, Figure C in Supplementary Material 5) were genetically closer to central-eastern Mediterranean wine varieties (sP2, predominantly assigned to proles *pontica* Figure D in Supplementary Material 5) than to other groups (Figure 3), and showed the highest divergence with the Central European group (sP6, assigned to proles *occidentalis* Figure H in Supplementary Material 5, Figure 3). It should be noted that sP3, according to NJ, comprises individuals from both *subproles caspica* and *antasiatica* within proles *orientalis* (Figure E in Supplementary Material 5), and these genotypes are mainly related to muscats, while those composing sP1 are mainly related to Hebén cv. (Supplementary Material 3). Bacilieri et al. [9] found that Iberian cvs. are genetically closer to eastern varieties than to Balkan ones, accordingly to Emanuelli et al. [10], who clustered Spanish varieties into the proles *orientalis*-*antasiatica*. In the same study, hierarchical STRUCTURE by SNPs clearly separated Spanish cvs. from proles *pontica*. On the contrary, in Laucou et al. [30], Spanish varieties showed the lowest pairwise F_ST_ with the Balkan group, which should be mainly composed of proles *pontica* and *orientalis*-*caspica* grapes [13]. Clustering methods and markers used cannot provide fully consistent outcomes anyway [9,24]. In any case, the hypothesis that Phoenicians and Greeks introduced grapevines to Spain belonging to proles *pontica* and *orientalis* [22,32] is always corroborated. When separating groups by country of origin, Spain shows the lowest F_ST_ versus Portugal (Figure 3), depending not only on geographical proximity but also on the partition of both accession pools into sP1, sP2 (mainly admixed) and sP5 (Supplementary Material 3), while similar F_ST_ values are shown for pairwise Spain–Italy and Spain–France.

Differences among groups account for 10% of the total genetic diversity in the strongly assigned accession subset (Q ≥ 0.78, Figure 3B). When eco-geographic origins referred to a less extended total area, this percentage tends to decrease [13,33], although a higher value was shown when comparing well-clustered wild and cultivated forms [26]. Genetic diversity within groups is almost totally due to the intraindividual allelic variation, pointing out the high grapevine heterozygosity [3]. However, in our germplasm collection, it seems that the scenario of a *vinifera* structure linked to a large complex pedigree with grape breeding restricted to a relatively small number of elite cultivars [34] has been further stressed by receiving preferentially selected genotypes throughout time. Extending AMOVA to admixed accessions, the extent of the genetic diversity decreases due to differentiation among sPs as well as F_ST_ from F statistics (Figure 3B), indicating a low probability of genetic drift depending on geographical separation, especially when only the subarea, including Italy, France, Spain and Portugal, is considered (Figure 3C). These results, together with the consistent admixture levels in each K (Supplementary Material 3) and the weak relationship between pairwise F_ST_ comparison values and eco-geographic distances (Figure 3), support that the structure of the modern grapevine population has been shaped by a long history of combination of natural hybridization, breeding, selection, human-mediated movements of seeds and cuttings and other factors, as was proposed by Bacilieri et al. [9].

### 3.3. Mediterranean Iberian Peninsula Genetic Pool

Myles et al. [34] found strong evidence that the cultivated grape originated in the Near East and spread from that area westwards. Central and Western European grapevine groups showed some degree of genetic relationship with Eastern “*sylvestris*” confirming an East-West gene flow by the movement of cultivated genotypes [34,35]. In addition, in these regions, secondary domestication events involving local wild forms took place [24,36,37]. Given that wild and cultivated populations showed very close genetic diversity, Myles et al. [34] also suggested that many cultivars in use today may only be a small number of generations removed from the wild progenitor and claimed that introgression occurred from Western *sylvestris* to Western *vinifera* but not vice versa. This may explain why some ancient Central European varieties (proles *occidentalis*), such as Clairette, Pinot Noir, etc., conserve clear wild morphological traits [27,32,35]. Meanwhile, Arroyo-García et al. [15] showed a great insertion of typical Eastern chlorotypes (especially C and D) in Italian, French and German varieties, although French and German sylvestris display nearly 100% chlorotype A. In fact, the majority of analyzed varieties from France and Germany/Austria are from migrated Heunisch (C) and Savagnin (D) and their offspring. Instead, in Spain, the huge majority of both commercial varieties and still conserved wild types displayed the type A. Thus, at first sight, chlorotype indications seem to disagree from genetic relationships based on nuclear Deoxyribonucleic Acid (DNA) markers, with the latter suggesting the Spanish germplasm to be at least as close to the Eastern genotypes as the Italian, French and German ones are. A possible scenario is deducible from De Andrés et al. [26]: Spanish wild grapevines are essentially divided in two groups, northern (NSW) and southern (SSW), the latest being the most genetically close to cultivated varieties. In Myles et al. [34], PCA separated the SSW group from other *sylvestris* populations that included NSW members.

Interestingly, Eastern cultivated grapes are closer to SSW than to other European *sylvestris*. Myles et al. could not prove a lack of introgression from Spanish cultivated grapes into SSW because of the very low number of Spanish cvs. included in that study. However, De Andrés et al. [26] detected a significant number of spontaneous *vinifera*-*sylvestris* hybrids in Southern wild populations, which could mean gene-flow occurred in both directions (for example, Zalema cv., which is very important in Andalusia, showed a very close relation to *sylvestris* genotypes). Therefore, since Eastern grapevines were introduced in Spain by Phoenician and Greeks, putative repeated hybridization and backcrossing events between both subspecies may be supposed, resulting in the reduction of the genetic diversity among them (more than in other European areas) and obtaining new domesticated forms, without discarding the possibility that some primordial domestication had occurred even in former times [38,39]. Throughout this complex process, some female domesticated vines appeared and its fertilization with pollen from imported cultivars originated hybrids with oriental phenotypes conserving chlorotype A, as in the case of Hebén cv., which is an ancestor of the majority of sP1 individuals and was shown previously to be a parent of several Spanish and Portuguese varieties [40]. Despite this hypothesis encloses some speculative elements, it is evident that sP1 accession pool originated by a consistent genetic contribution from oriental grapevines and a long-time interaction between wild and cultivated forms. Andalusia has surely represented a pivotal center of biodiversity development given that this region holds the main reservoir of Southern Spain wild vine populations [41] and Hebén, which was first described by Clemente and Rubio [42], has been cultivated in several areas within and close to Andalusia since very long time [21]. To delve into the question, determining the parents of Hebén, as well as the origin of other chlorotype A varieties, would be extremely helpful, given that it is a very hard issue, often depending on lucky archaeobotanical findings [3]. Likewise, the discovery of the origin of Garnacha, which fits in sP1 and is a parent of some French varieties included in this cluster, would further clarify grape domestication and evolution in Western Europe.

Finally, it is worth mentioning that Eastern genotypes’ contribution to Spanish grapevines is additionally proven by the presence of some accessions carrying the chlorotype D, such as Palomino Fino, the main wine variety in Andalusia, and Jaén Tinto and Doradilla, which are considered autochthonous of this region. Indeed, the type D chlorotype is common in wild forms eastward from Italy to the Middle East [15] and its presence in Spain was previously discussed [23].

### 3.4. Core Collections

Core-35 and -63 include 7.3% and 13.1% of the total accessions of the collection, respectively, these results being in accordance with previous studies [43]. These cores are consistent because they include an acceptable percentage of each cluster inferred by structure analysis (Table 5). Therefore, they may be suitable for future association studies or at least provide an idea about the optimal size and cluster composition. However, when a specific study will be engaged, the real objective of the working core collection must be carefully analyzed, and consequently, some additional questions should be taken into account: (a) the possibility of including phenotypic traits of agronomical and/or commercial interest, (b) the chance of genotype-phenotype covariance due to individual relatedness, which should be avoided by removing/substituting some accessions [44] and (c) the possibility of including a priori in the kernel file of MSTRAT some key varieties (e.g., Pinot Noir, Merlot and Sangiovese), which are excluded by the present analysis. Finally, we remark the presence in both cores of individuals from sP7, given that only four accessions within this cluster are putative *Vitis vinifera*.

## 4. Materials and Methods

### 4.1. Plant Material

The grapevine germplasm repository “Rancho de la Merced” is located at the namesake IFAPA center, occupying approximately a 3.5 ha surface area (google maps location link: https://www.google.es/maps/place/IFAPA+Center+Rancho+la+Merced/@36.72794,6.1658487,17z/data=!3m1!4b1!4m5!3m4!1s0xd0dc3fd6b941443:0x6e5d06.16366 (accessed on 12 April 2022)). It preserves about 1800 accessions, including *Vitis vinifera* subp. *vinifera* and *Vitis vinifera* subp. *sylvestris*, other *Vitis* species, RS and IC (or hybrids direct producers, commonly known as HPD,). Each accession is in a subplot consisting of five biological replicates obtained by grafting scions on RS 161-49 Couderc. For most of the accessions, the following data are available: accession name, country and center that provided cuttings, year of reception, phenology (budburst, flowering, veraison and harvest), must quality (yield, pruning weight, Baumé and titratable acidity), use and skin Page 7/16 color [16]. In this paper we studied all accessions maintained in the section designated to conserve *Vitis vinifera* wine grape varieties, composed of 930 subplots. Two vines out of five replicates per accession were analyzed, although in some cases, only one plant was available.

### 4.2. Microsatellite Analysis

DNA was extracted from 0.05 g of fresh young leaves using the DNeasy 96 Plant Kit (Qiagen, Düsseldorf, Germany) with a slight modification consisting in the addition of a pinch of polyvinylpyrrolidone PVP 40,000 to the extraction buffer. DNA yield and quality were determined by NanoDrop ND-1000 spectrophotometer (NanoDrop Technologies, Wilmington, USA). In some cases, quality was further checked in 1% agarose gels after RedSafeTM staining under UV light and, for poor quality DNA samples, re-extraction was performed by DNeasy Plant Mini Kit (Qiagen, Düsseldorf, Germany) on 0.1 g young leaves frozen in liquid nitrogen. Thirteen SSR markers were analyzed, i.e., the nine included in the GrapeGen06 set (see introduction) and four others (ISV2, ISV3, ISV4 and VMCNG4b9) that are routinely used at the “Consiglio per la Ricerca in Agricoltura e l’analisi dell’Economia Agraria-Uva da Tavola” (CREA-UTV, Turi, Italia, [45]). Four multiplex PCRs were set up in a 10 μL vol containing less than 50 ng of DNA, 5 pmol of each forward and reverse primer and 5 μL of DNA Amplitools Master Mix (Biotools, Madrid, Spain). Forward primers were labeled with WellRED dyes D2-PA, D3-PA or D4-PA (Sigma-Aldrich, San Luis, USA) at the 5′ end. The cycling profile consisted of an initial heat activation step at 96 °C for 3 min, 36 cycles of denaturation at 94 °C for 20 s, 30 s annealing at temperatures ranging from 56 to 63 °C depending on the lowest primer melting temperature, extension at 72 °C for 50 s and a final extension step at 72 °C for 15 min. Amplicons were separated on a GenomeLabTM GeXP Genetic Analysis System (Beckman Coulter, Brea, USA) and sized using the manufacturer’s software. Alleles occurring less than six times were carefully checked by electropherogram visual inspection and errors were corrected accordingly. Profiles of reference varieties Garnacha, Merlot, Shiraz and Gewürztraminer were used to harmonize SSR allele sizes and compare genotypes with the VIVC. Profile comparison was extended to other *Vitis* databases when no match was found in VIVC: the Grapevine Collection at the FEM (ITA362), the Germplasm Repository at the “Consiglio per la Ricerca in Agricoltura e l’analisi dell’Economia Agraria-Viticoltura ed Enologia” (CREA-VIT, Conegliano, ITA388, [46]), the Italian *Vitis* Database (http://www.Vitisdb.it/ (accessed on 12 April 2022)), The “Instituto Madrileño de Investigación y Desarrollo Rural, Agrario y Alimentario” (IMIDRA) Grapevine Germplasm Collection (ESP080, https://www.comunidad.madrid/info/coleccion-vid?nombre_local=palomino&nombre_principal=&nombre_local_exact=&nombre_principal_exact= (accessed on 12 April 2022)), the Italian Grapevine Variety National Register (http://catalogoviti.politicheagricole.it/result.php?codice=315 (accessed on 12 April 2022)), the variety collection at Canarias Islands (http://Vitiscanarias.com/ (accessed on 12 April 2022)) and the Grapevine Variety Collection at the “Instituto de la Vid y el Vino de Castilla la Mancha” (IVICAM, ESP216 http://pagina.jccm.es/ivicam/servicios/microsatelites/microsatelites.php (accessed on 12 April 2022)). In addition, for the GrapeGen06 SSR set, data were coded for comparability of microsatellite profiles according to Maul et al. [47] in order to be included in the European *Vitis* Database (http://www.eu-Vitis.de/index.php (accessed on 12 April 2022)).

### 4.3. Analysis of the Data

The main variety name, the use and the country of origin to each identified genotype was assigned according to the information given on the databases consulted (see above), mainly VIVC. Non-matching genotypes as well as missing information were annotated as “unidentified”. The genetic grouping of the germplasm under study—considering just the unique genetic profiles detected—was explored through different statistical methods.

A Bayesian clustering algorithm to sort individuals into K clusters (subpopulations) according to their genetic similarity was performed using STRUCTURE 2.3.4 [48]. The best K is chosen based on the estimated membership coefficients (Q) for each individual in each cluster. Ten independent runs for K values ranging from 1 to 15 were performed with a burn-in length of 500,000 followed by 750,000 iterations. The admixture model with correlated allele frequencies was assumed and no prior population information was set up. The membership coefficient threshold defined for individual assignment to a given cluster was Q = 0.78. STRUCTURE HARVESTER 0.6.93 [49] was used for visualizing STRUCTURE output and inferring the most likely subdivision (K) by: (a) plotting the log probability L(K) following the plateau criterion proposed by Pritchard and Falush [50] and (b) by ΔK method according Evanno et al. [51]. Additional data analysis and formatting was subsequently applied to STRUCTURE results with CLUMPP [52] and DISTRUCT [53]. CLUMPP permutes the clusters output by the 10 independent runs of STRUCTURE, so that the clusters align across runs, while DISTRUCT allows the graphical representation of the aligned cluster assignments for a single K value. CLUMPAK [54] was used to align single results obtained for different K values (2, 3, 5 and 7). These single results consisted of averages obtained with CLUMPP for multiple independent runs.

Data were also evaluated using the Poppr package [55] in R (3.1.3 version, https://www.r-project.org (accessed on 12 April 2022)). Initially, the SSR dataset was examined by using the discriminant analysis of principal components (DAPC) implemented in the Adegenet package ver. 2.0.1 [56,57]. Prior clusters were identified by a sequential K-means clustering algorithm (find.clusters function) after data transformation by principal component analysis (PCA). Then, a discriminant analysis (DA) used part of the principal components (PCs) to describe the clusters. K-means was run with K varying from 1 to 20 and to ensure convergence, we increased the number of starting points to 200. The number of clusters was chosen based on the Bayesian Information Criterion (BIC) [58]. In order to avoid retaining too many dimensions at the DA step, the optimal number of PCs was computed by using both “optim.a.score” and “xvalDapc” functions from “adegenet”. The final cluster assignment was obtained after the DA analysis.

Moreover, an unweighted neighbor-joining (NJ) tree was constructed based on the Simple Matching dissimilarity index (SM) between the unique genetic profiles using Darwin software package v6.0 [59]. One thousand bootstrap replicates were performed. A further cluster analysis with Darwin was performed, including 101 varieties with a clear ancestry inferred by Emanuelli et al. [10] in accordance with the eco-geographic origin of the cultivars [22]. In this case, only the nine SSR markers analyzed in common for both datasets were considered (VVS2, VVMD5, VVMD7, VVMD25, VVMD27, VVMD28, VVMD32, VrZAG62 and VrZAG79). The germplasm from the Iberian Peninsula was further explored by both STRUCTURE and DAPC analysis, as described above. Genotypes from Spain and Portugal were assigned to a geographic area of origin and/or cultivation within the Iberian Peninsula corresponding to six pre-stablished areas: north-west (NW), north-east (NE), central-west (CW), central-east (CE), south-west (SW), south-east (SE), CAN (Canary Islands) and BAL (Balearic Islands).

Genetic diversity analyses were conducted on the pool of *Vitis vinifera* genotypes. Following statistics were determined with GenAlEx 6.0 [60]: number of alleles (Na), mean number of alleles per locus (MNA), effective number of alleles (Ne), observed heterozygosity (Ho), expected heterozygosity (He), Fixation Index (F), also called inbreeding coefficient, and the probability of identity (PI). The Excel add-in Microsatellite Toolkit [61] was used to determine the polymorphism information content (PIC). All these parameters were obtained for: (a) the overall *Vitis vinifera* population; (b) for the main country groups according to VIVC information; (c) for each cluster pointed out by STRUCTURE. We also studied the hierarchical genetic variation among and within inferred subpopulations by AMOVA and their genetic differentiation by the F-statistic (F_IS_, F_ST_ and F_IT_, where _I_ means individuals, _S_ subpopulations and _T_ the total population), including the calculation of F_ST_ for each group pairwise comparison.

Two genetic core collections of the *vinifera* set were constructed using the M (maximization) method, suggested by Schoen and Brown [62] and implemented in MSTRAT [63]. In the former, rare alleles (less than 1% frequency) were discarded; in the latter, all alleles were considered while fixing in the kernel file all accessions needed for the former. The redundancy step was previously performed to have an indication about the core collection size (n), setting 20 replicates, 200 iterations and Nei index as criterion of maximization. Then, core constructions were obtained testing different putative core size setting 100 replicates and 200 iterations. The final size was the lowest “n” that means the corresponding most represented “n” genotypes within the 100 replicates were able to capture all allele richness searched.

## 5. Conclusions

The genetic characterization of this grapevine collection conserved at Rancho de la Merced has allowed to identify mislabeling and redundant accessions, somatic and molecular variants, as well as new grapevine genetic resources. These results will help to optimize the management of one of the most important international grapevine germplasm repositories. In addition, the information obtained could represent an important updating of the VIVC. A consistent genetic diversity has been revealed in both the full *Vitis vinifera* pool and the clusters inferred by structure analysis. Clustering of samples at k = 2 discriminates grapes cultivated in a Mediterranean climate from Continental-Oceanic ones, while k = 7 infers more restricted ecogeographic areas of cultivation. A core collection capturing allele richness may be suitable for association studies given that an equilibrated accession contribution from each of the six main eco-geographic inferred groups was achieved. Varieties from the Iberian Peninsula cultivated under Mediterranean conditions form a quite genetically homogenous group and, in accordance with previous studies, our results confirm that the origin of many Spanish (and Andalusian) varieties is strongly related to both local *Vitis sylvestris* and Eastern *Vitis vinifera* cultivars introduced in Spain by trade.

## Figures and Tables

**Table 1:** Accession and genotype categorization.

Accessions								Genotypes								
Identified						Unidentified	Total analyzed	SSR genotypes represented by only one accession	SSR genotypes represented by two or more accessions	Different SSR genotypes						
Correct accession name	Verified synonymies *	Not verified synonymies	Without accession name	Wrong accession name	Total					Total	Wine	Dual-use	Table	Interbreeding cross	Rootstock	Undefined
264	358	101	43	125	891	46	937	350	587	521	297	121	29	24	4	46

* Most are reported in the *Vitis* International Variety Catalogue (VIVC), other are indicated by Cabello et al. [21] or in commercial variety catalogues (https://www.mapa.gob.es/app/regVar/ (accessed on 12 April 2022)).

**Table 2:** Distribution of varieties assigned at K = 7 in the other STRUCTURE grouping levels and Discriminant Analysis of Principal Components (DAPC).

**STRUCTURE K = 7**	**Genotypes with ancestry (Q) > 0.78**										**Admixed**
	group	**sP1**	**sP2**	**sP3**	**sP4**	**sP5**	**sP6**	**sP7**	Total		
	genotypes	73	37	42	35	51	23	23	284		237
		**Assigned in other STRUCTURE K and DAPC**								**Not assigned** **(Q < 0,78)**	
									Total		
**STRUCTURE Q** **≥0.78**	**K = 2**	73/0	23/0	9/7	0/30	2/26	0/20	0/17	207	77	
	**K = 3**	72/0/0	8/1/0	0/33/0	0/0/16	0/0/42	0/0/14	0/21/0	207	77	
	**K = 5**	73/0/0/0/0	0/29/0/0/0	0/0/41/0/0	0/4/2/2/0	0/0/0/48/0	0/0/0/1/17	0/0/0/0/22	239	45	
**STRUCTURE** **Greater Q value**	**K = 2**	73/0	37/0	22/20	0/35	5/46	1/22	0/23	284		
	**K = 3**	73/0/0	25/12/0	1/41/0	0/10/25	3/0/48	0/1/22	0/23/0	284		
	**K = 5**	73/0/0/0/0	1/36/0/0/0	0/0/42/0/0	0/15/6/12/2	0/0/0/51/0	0/0/0/1/22	0/0/0/0/23	284		
**DAPC**											**K=7 admixed allocation in DAPC groups**
	**DAPC = 2**	28/45	19/18	32/10	29/6	41/10	13/10	8/15			71/166
	**DAPC = 3**	0/5/68	1/20/16	0/36/6	15/20/0	47/2/2	22/1/0	3/19/1			79/89/69
	**DAPC = 5**	1/1/67/4/0	0/37/0/0	40/1/0/1/0	1/2/0/23/9	0/5/0/7/39	0/1/0/1/21	4/2/0/14/3			31/67/35/50/54

sP means subpopulation (see Figure 1); Q: membership coefficient; K: number of clusters set by STRUCTURE. Admixed is referred to individuals not clear belonging to any group (the highest Q is ≤ 0.78).

**Table 3:** Main polymorphism indicators for each microsatellite.

Locus	All Accessions (521)			*Vitis vinifera* (481)		
	PIC	N	<1%	PIC	N	<1%
MD7	0.790	17	6	0.772	13	4
MD32	0.794	13	5	0.796	11	3
ZAG62	0.794	15	6	0.786	10	2
ZAG79	0.841	14	3	0.830	12	1
EVA2	0.854	21	10	0.849	17	6
ISV2	0.842	23	13	0.829	18	10
VVS2	0.823	17	6	0.819	15	4
MD5	0.844	16	7	0.839	10	1
MD27	0.811	15	7	0.806	10	2
MD25	0.734	14	9	0.730	12	7
MD28	0.865	22	11	0.858	18	7
ISV4	0.786	13	6	0.780	12	5
ISV3	0.626	13	7	0.623	10	4

PIC: Polymorphic Index Content; N: number of different alleles; <1%: number of rare alleles with frequency less than 1%.

**Table 4:** Diversity indices calculated for different sets of genotypes from data of 13 nuclear microsatellite loci.

Sample		N	Na	Ne	Ho	He	F	PI
Most represented countries	Spain	82	9.15	5.08	0.829	0.790	−0.053	6.02 × 10^−16^
	Portugal	98	9.38	5.52	0.852	0.809	−0.054	8.10 × 10^−17^
	Italy	57	8.31	5.02	0.835	0.785	−0.065	1.27 × 10^−15^
	France	113	9.08	4.80	0.855	0.779	−0.102	2.37 × 10^−15^
	Total	350	11.31	5.64	0.845	0.813	−0.042	5.38 × 10 ^−17^
Inferred by STRUCTURE according to Q ≥ 0.78	sP1	73	6.92	3.97	0.828	0.728	−0.143	2.13 × 10 ^−13^
	sP2	37	7.85	4.65	0.834	0.770	−0.085	5.03 × 10 ^−15^
	sP3	42	7.08	3.95	0.799	0.730	−0.101	1.72 × 10 ^−13^
	sP4	35	7.77	4.51	0.829	0.765	−0.087	9.78 × 10 ^−15^
	sP5	51	7.38	4.35	0.849	0.754	−0.126	2.40 × 10 ^−14^
	sP6	21	4.77	3.45	0.817	0.695	−0.176	6.21 × 10 ^−12^
	Total	258	11.08	5.76	0.828	0.816	−0.019	3.61 × 10 ^−17^
Inferred by STRUCTURE according to the highest Q	sP1	110	9.08	4.43	0.841	0.754	−0.119	1.84 × 10 ^−14^
	sP2	90	9.62	5.14	0.839	0.795	−0.058	4.54 × 10 ^−16^
	sP3	70	8.62	4.52	0.825	0.764	−0.086	8.20 × 10 ^−15^
	sP4	78	8.85	4.90	0.825	0.785	−0.055	1.13 × 10 ^−15^
	sP5	85	9.23	4.77	0.851	0.775	−0.098	3.01 × 10 ^−15^
	sP6	44	7.23	4.21	0.818	0.743	−0.102	6.75 × 10 ^−14^
	Total	477	12.62	5.78	0.835	0.817	−0.025	3.01 × 10 ^−17^
All Vitis vinifera *		481	12.92	5.79	0.835	0.818	−0.025	2.91 × 10 ^−17^

sP means subpopulation (see Figure 1). Q: membership coefficient, N: number of varieties/accessions, Na: mean number of alleles per locus, Ne: effective number of alleles per locus, Ho: observed heterozygosity, He: expected heterozygosity, F: fixation index, PI: cumulative probability of identity. * Four genotypes from sP7 are included (see STRUCTURE analysis).

**Table 5:** Contribution to core collections of each group inferred by STRUCTURE (k = 7) as number of accessions.

		Core Collections	
		Core-35 (No Rare Alleles)	Core-63 (All Alleles)
sP1	Q ≥ 0.78	3	6
	admix	5	8
	sP percent	7.27%	12.72%
	**Core percent**	**22.85%**	**22.22%**
sP2	Q ≥ 0.78	2	6
	admix	1	4
	sP percent	3.33%	11.11%
	**Core percent**	**8.58%**	**15.87%**
sP3	Q ≥ 0.78	4	5
	admix	2	4
	sP percent	8.57%	12.85%
	**Core percent**	**17.14%**	**14.29%**
sP4	Q ≥ 0.78	4	6
	admix	3	4
	sP percent	8.97%	12.82%
	**Core percent**	**20%**	**15.87%**
sP5	Q ≥ 0.78	4	5
	admix	3	8
	sP percent	8.23%	15.29%
	**Core percent**	**20%**	**20.63%**
sP6	Q ≥ 0.78	-	-
	admix	3	4
	sP percent	6.81%	9.09%
	**Core percent**	**8.58%**	**7.35%**
sP7	Q ≥ 0.78	-	-
	admix	1	3
	sP percent	25%	75%
	**Core percent**	**2.85%**	**4.76%**
He		0.825	0.833
Ho		0.873	0.840

sP 1–7: see Figure 1. Ho: observed heterozygosity; He: expected heterozygosity; Q: membership coefficient; admix indicate individuals assigned to a given sP based on the highest Q (≤ 0.78).

## Data Availability

The datasets generated during the current study are not publicly available yet. Full accession information is stored among the informatic resources of the IFAPA center “Rancho de la Merced” and the authors will be glad to provide any data required for research purposes.

## Supplementary Materials

The following supporting information can be downloaded at: https://www.mdpi.com/article/10.3390/plants11081088/s1. Supplementary Material 1: Genotypic profiles and additional information of all accessions studied [64,65]; Supplementary Material 2: Estimated number of clusters obtained with STRUCTURE for K values from 1 to 15 using 13 SSR. (a) Graphical representation of estimated mean L(K). (b) Graphical representation of ΔK statistics. (c) Table summarizing parameters of different STRUCTURE simulations performed for each preset K: mean likelihoods of models and ΔK; Supplementary Material 3: Cluster assignation of unique genotypes by STRUCTURE analysis and core collections genotypes; Supplementary Material 4: DAPC clusters; Supplementary Material 5: Neighbor-joining dendrograms. Figures A and B, based on simple matching dissimilarity matrix calculated from the dataset of 13 SSRs across the 521 unique genotype collection and the 258 accessions belonging to the six Vitis vinifera subpopulations inferred by STRUCTURE. Figures C–H, based on 9 OIV SSR datasets, including 101 cultivars from FEM collection with clear eco-geographic origin (Emanuelli et al. [10]) and 230 accessions from this study belonging to the six Vitis vinifera subpopulations inferred by STRUCTURE. Figure I, putative geographical representation of sPs inferred by STRUCTURE in this study; Supplementary Material 6: DAPC analysis on the Iberian germplasm.
