# Supplementary material for: Genetic Characterization by SSR Markers of a Comprehensive Wine Grape Collection Conserved at Rancho de la Merced (Andalusia, Spain)
Source: Plants (Basel). 2022 Apr 16;11(8):1088. doi: 10.3390/plants11081088 (PMC9028831; doi:10.3390/plants11081088)
Supplement: Supplementary file 1 [file plants-11-01088-s001.zip › Supplementary Material 5.pptx]

## Slide 1
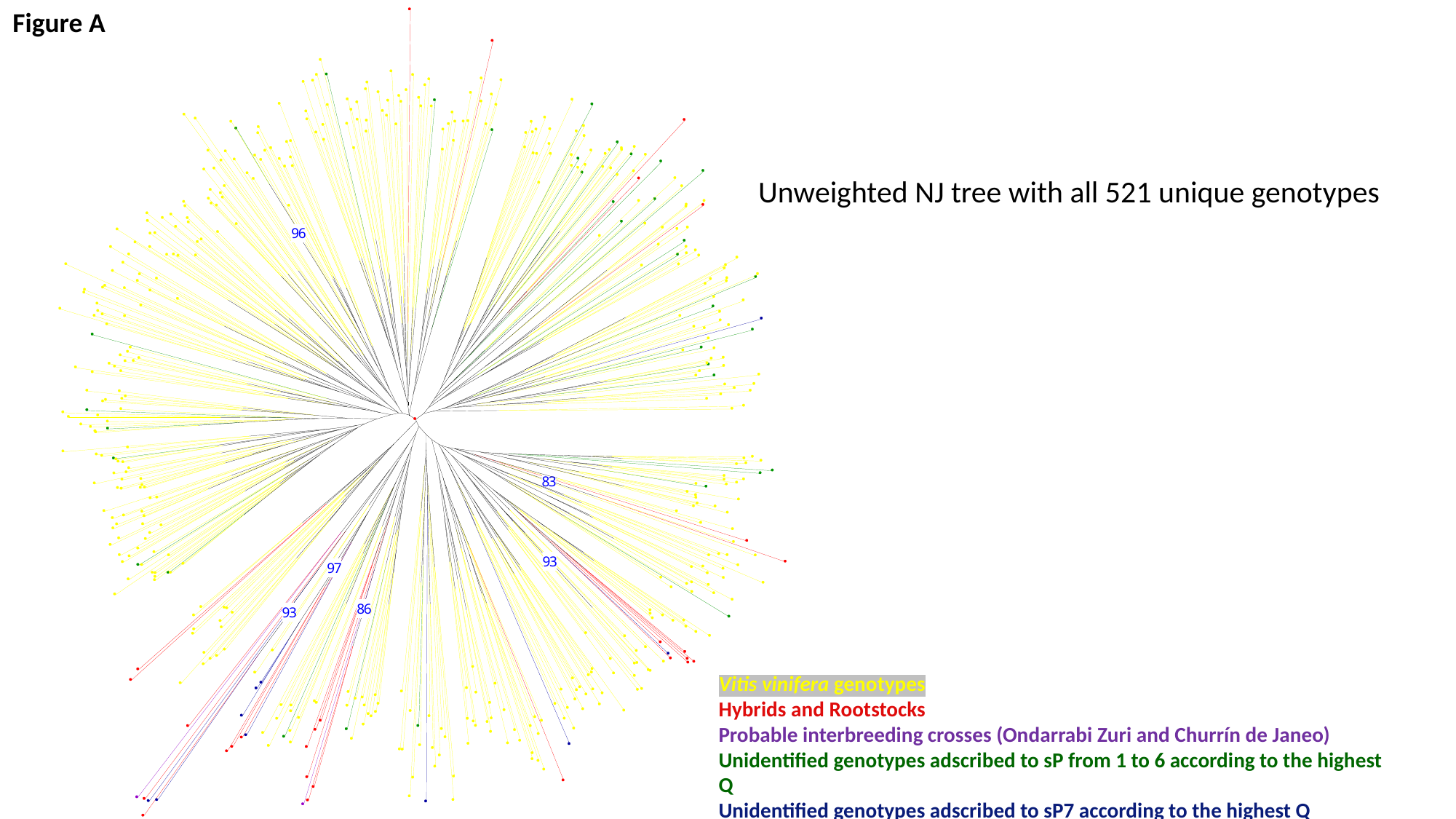

Figure A
Unweighted NJ tree with all 521 unique genotypes
Vitis vinifera genotypes
Hybrids and Rootstocks
Probable interbreeding crosses (Ondarrabi Zuri and Churrín de Janeo)
Unidentified genotypes adscribed to sP from 1 to 6 according to the highest Q
Unidentified genotypes adscribed to sP7 according to the highest Q

## Slide 2
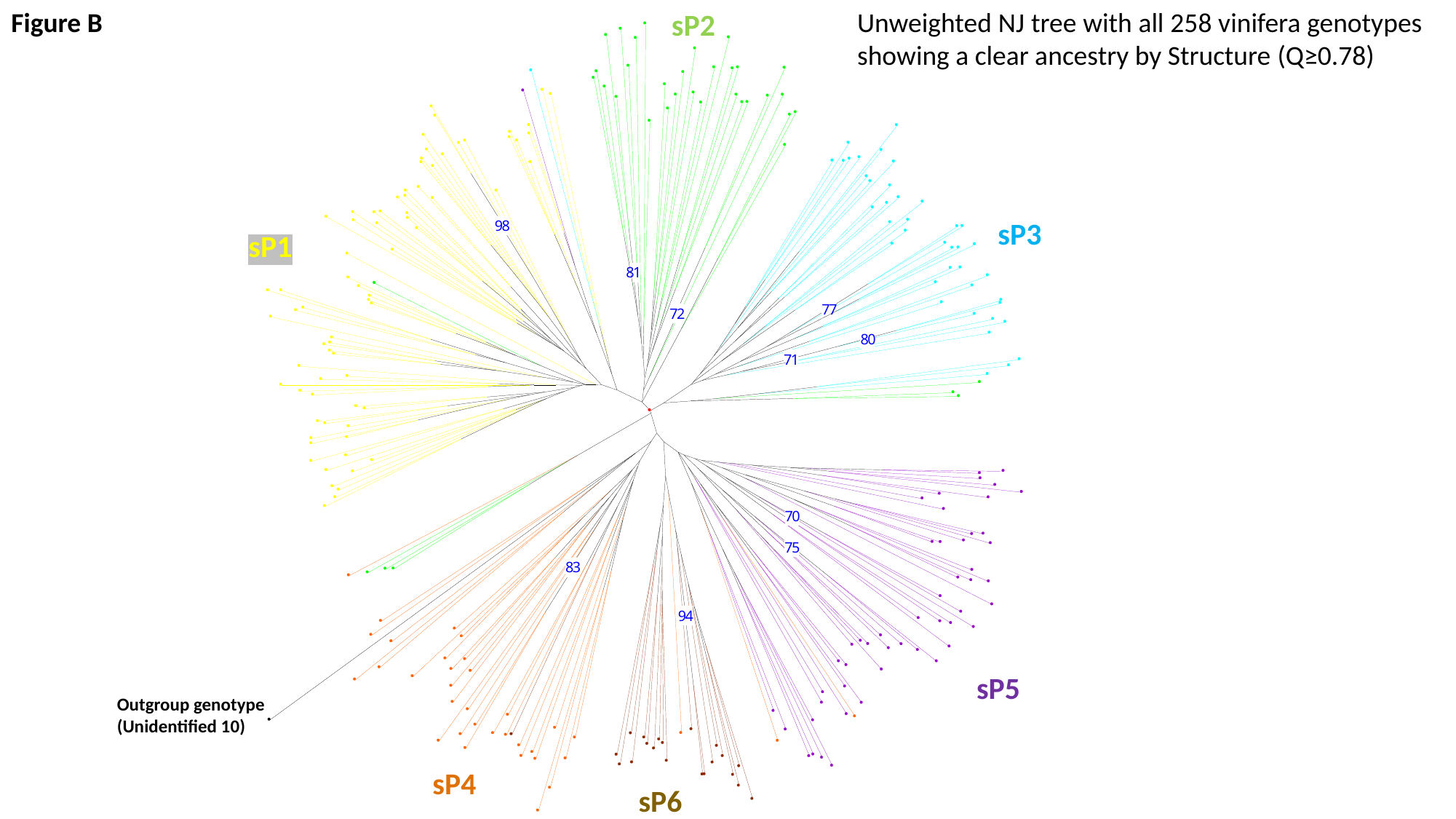

Figure B
sP2
Unweighted NJ tree with all 258 vinifera genotypes showing a clear ancestry by Structure (Q≥0.78)
sP3
sP1
sP5
Outgroup genotype (Unidentified 10)
sP4
sP6

## Slide 3
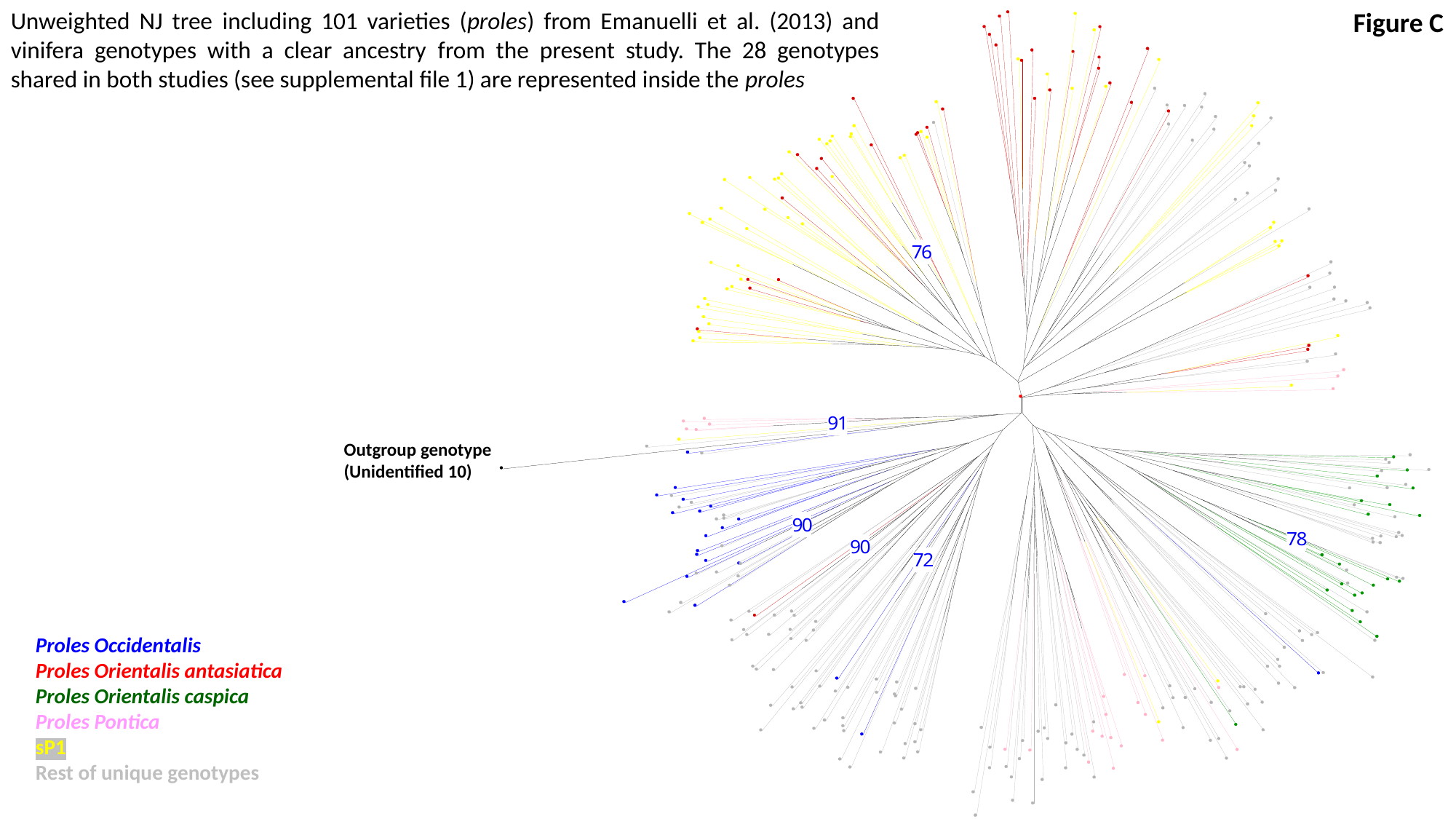

Unweighted NJ tree including 101 varieties (proles) from Emanuelli et al. (2013) and vinifera genotypes with a clear ancestry from the present study. The 28 genotypes shared in both studies (see supplemental file 1) are represented inside the proles
Figure C
Outgroup genotype (Unidentified 10)
Proles Occidentalis
Proles Orientalis antasiatica
Proles Orientalis caspica
Proles Pontica
sP1
Rest of unique genotypes

## Slide 4
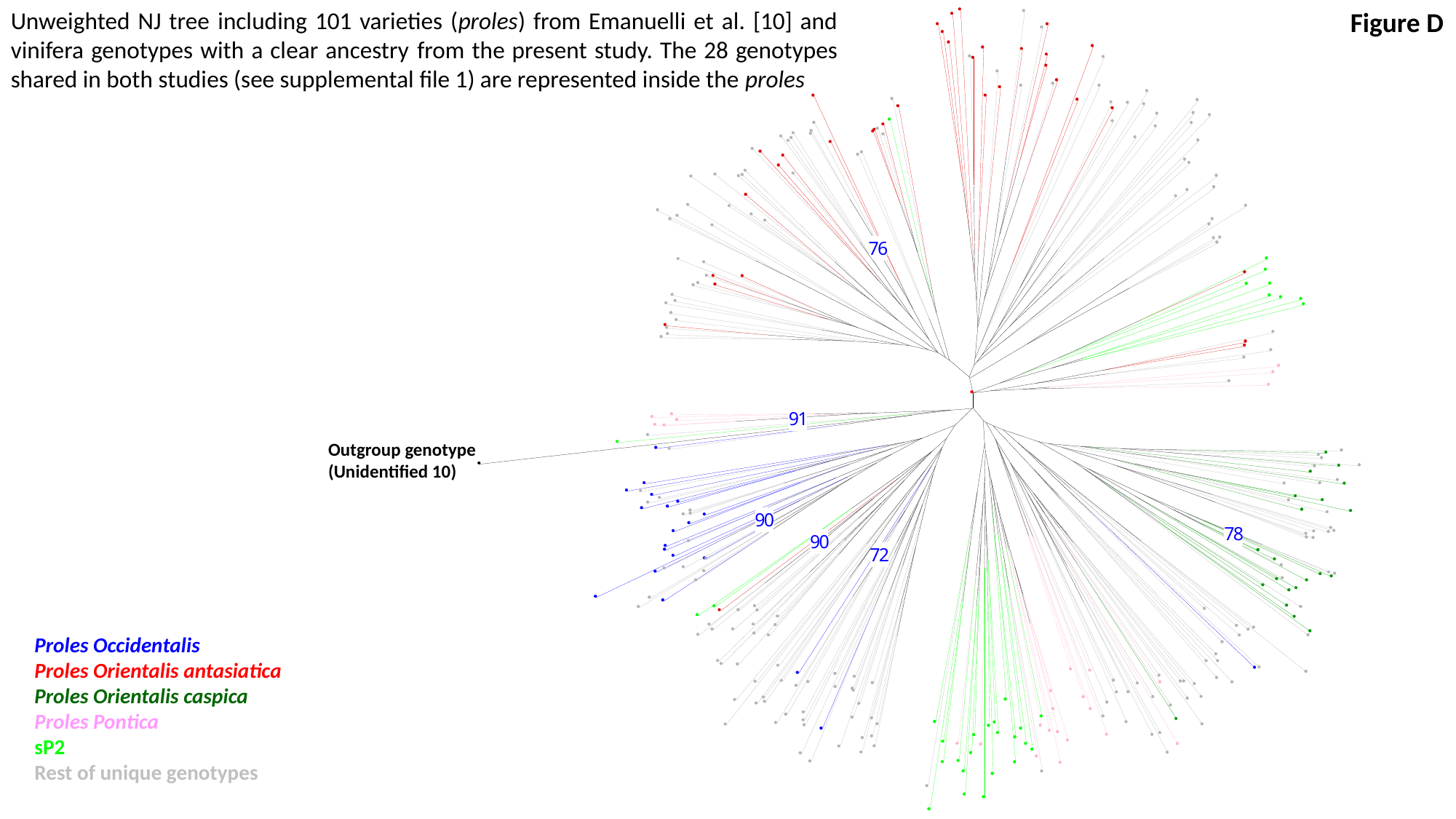

Unweighted NJ tree including 101 varieties (proles) from Emanuelli et al. [10] and vinifera genotypes with a clear ancestry from the present study. The 28 genotypes shared in both studies (see supplemental file 1) are represented inside the proles
Figure D
Outgroup genotype (Unidentified 10)
Proles Occidentalis
Proles Orientalis antasiatica
Proles Orientalis caspica
Proles Pontica
sP2
Rest of unique genotypes

## Slide 5
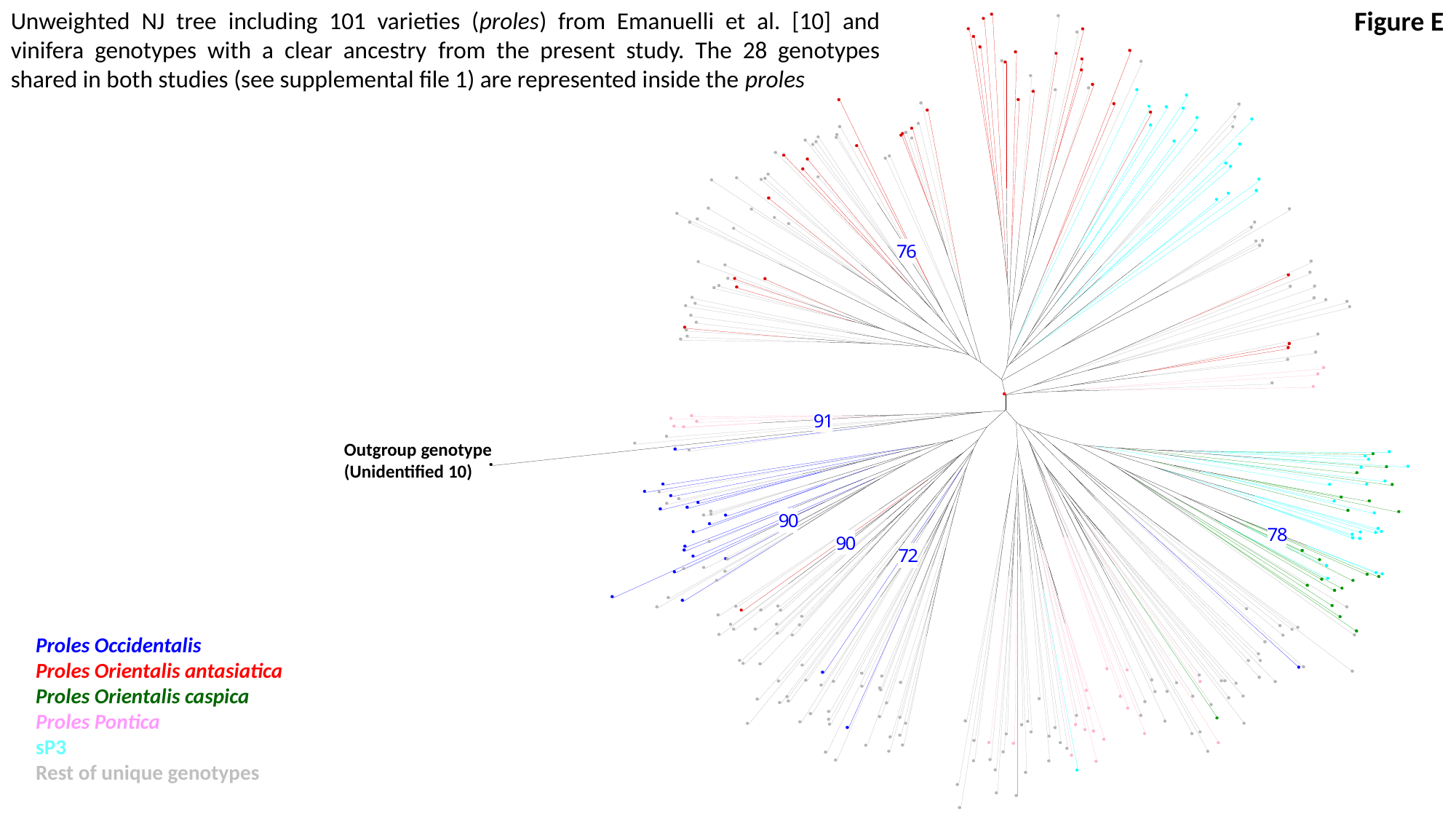

Unweighted NJ tree including 101 varieties (proles) from Emanuelli et al. [10] and vinifera genotypes with a clear ancestry from the present study. The 28 genotypes shared in both studies (see supplemental file 1) are represented inside the proles
Figure E
Outgroup genotype (Unidentified 10)
Proles Occidentalis
Proles Orientalis antasiatica
Proles Orientalis caspica
Proles Pontica
sP3
Rest of unique genotypes

## Slide 6
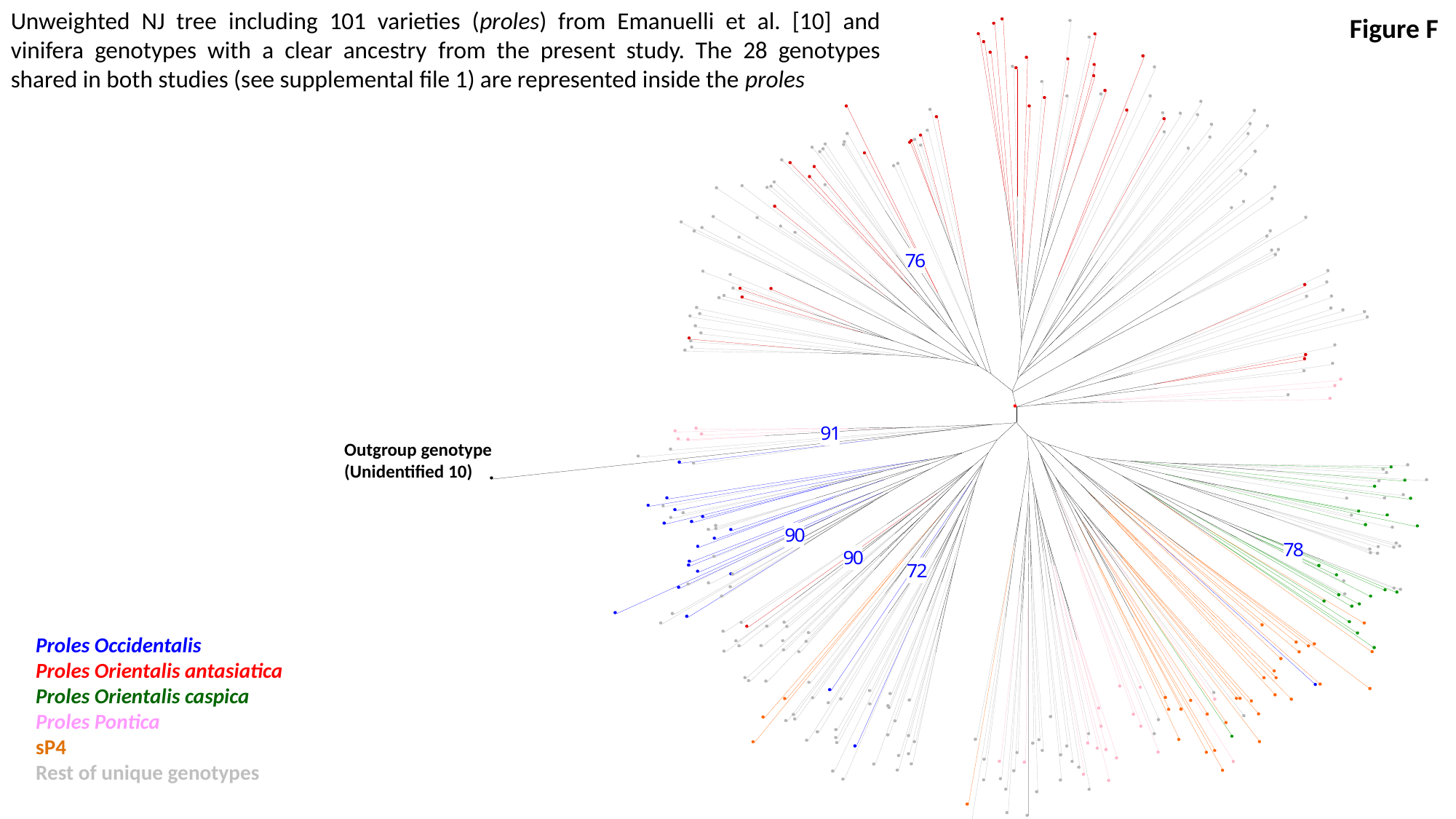

Unweighted NJ tree including 101 varieties (proles) from Emanuelli et al. [10] and vinifera genotypes with a clear ancestry from the present study. The 28 genotypes shared in both studies (see supplemental file 1) are represented inside the proles
Figure F
Outgroup genotype (Unidentified 10)
Proles Occidentalis
Proles Orientalis antasiatica
Proles Orientalis caspica
Proles Pontica
sP4
Rest of unique genotypes

## Slide 7
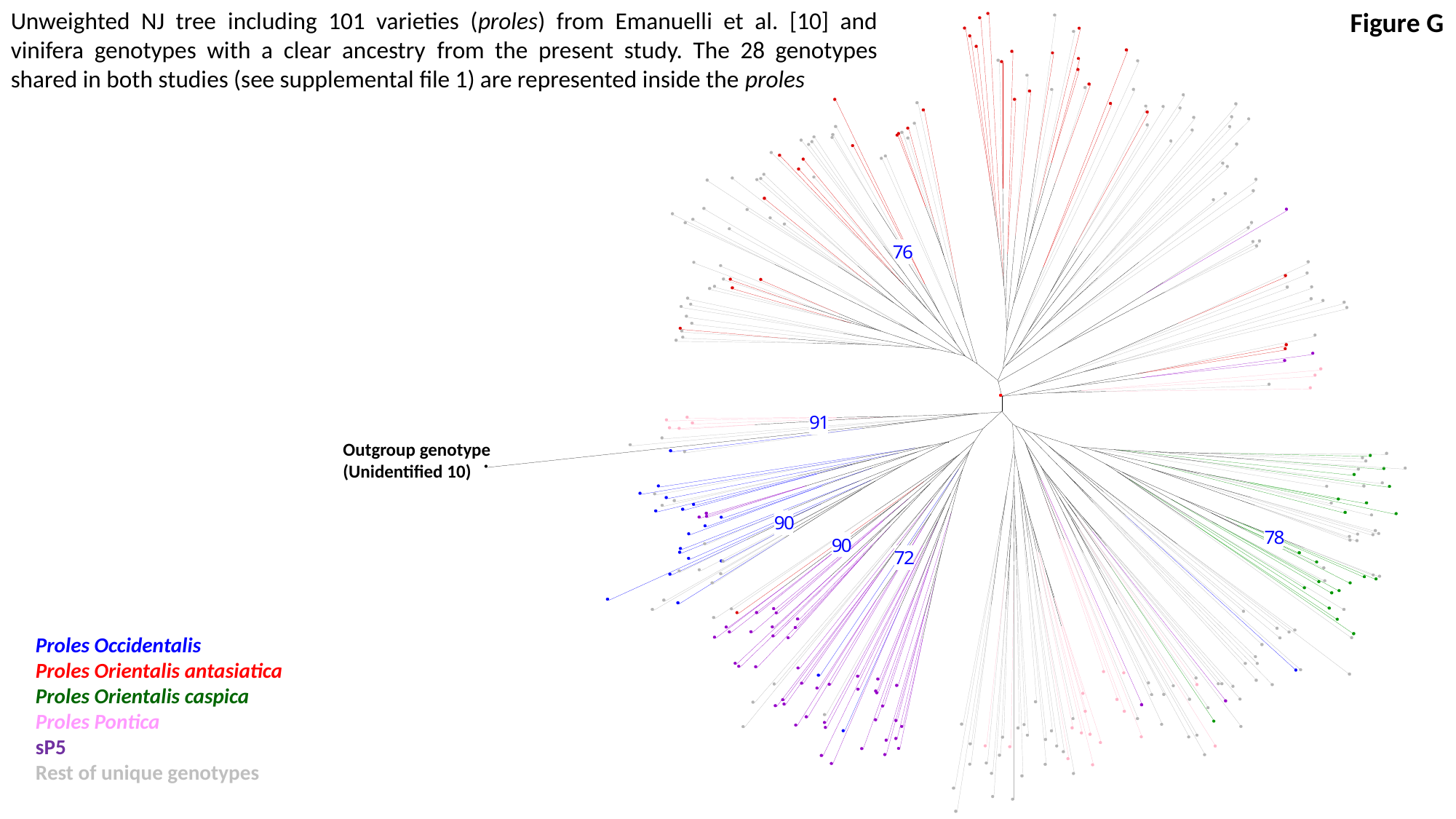

Unweighted NJ tree including 101 varieties (proles) from Emanuelli et al. [10] and vinifera genotypes with a clear ancestry from the present study. The 28 genotypes shared in both studies (see supplemental file 1) are represented inside the proles
Figure G
Outgroup genotype (Unidentified 10)
Proles Occidentalis
Proles Orientalis antasiatica
Proles Orientalis caspica
Proles Pontica
sP5
Rest of unique genotypes

## Slide 8
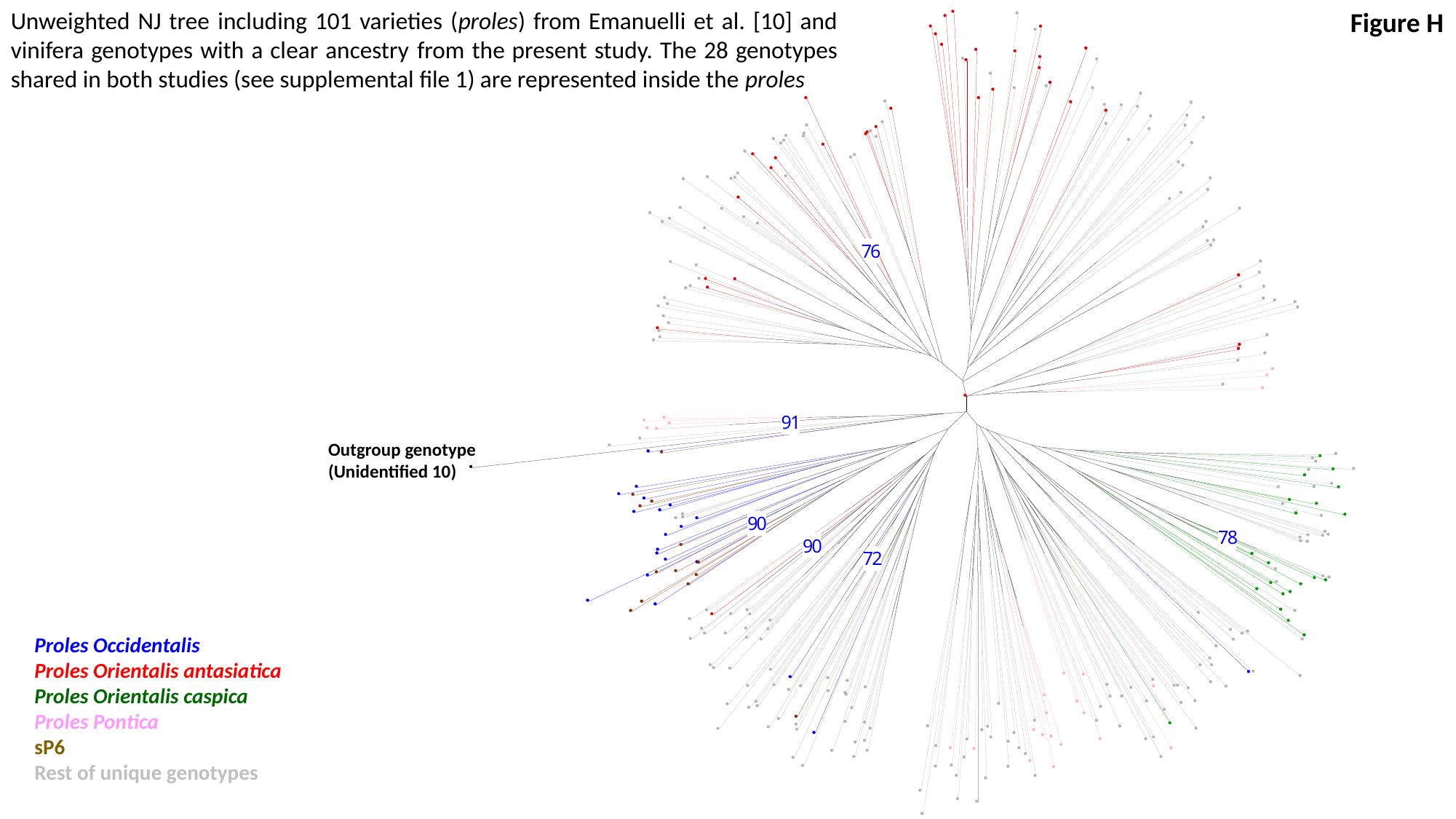

Unweighted NJ tree including 101 varieties (proles) from Emanuelli et al. [10] and vinifera genotypes with a clear ancestry from the present study. The 28 genotypes shared in both studies (see supplemental file 1) are represented inside the proles
Figure H
Outgroup genotype (Unidentified 10)
Proles Occidentalis
Proles Orientalis antasiatica
Proles Orientalis caspica
Proles Pontica
sP6
Rest of unique genotypes

## Slide 9
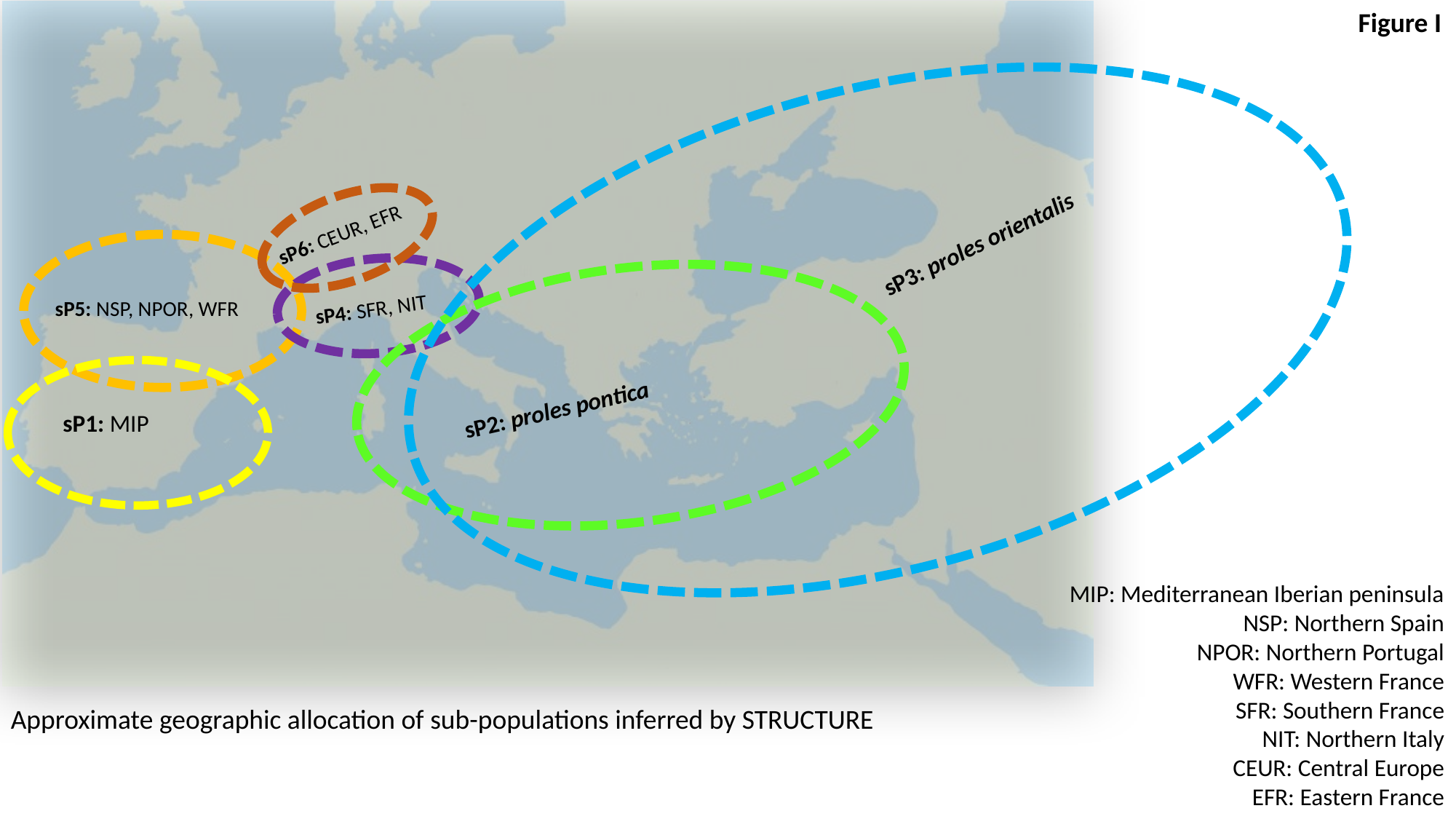

Figure I
sP3: proles orientalis
sP6: CEUR, EFR
sP5: NSP, NPOR, WFR
sP4: SFR, NIT
sP2: proles pontica
sP1: MIP
MIP: Mediterranean Iberian peninsula
NSP: Northern Spain
NPOR: Northern Portugal
WFR: Western France
SFR: Southern France
NIT: Northern Italy
CEUR: Central Europe
EFR: Eastern France
Approximate geographic allocation of sub-populations inferred by STRUCTURE
