# Supplementary material for: Genetic Characterization by SSR Markers of a Comprehensive Wine Grape Collection Conserved at Rancho de la Merced (Andalusia, Spain)
Source: Plants (Basel). 2022 Apr 16;11(8):1088. doi: 10.3390/plants11081088 (PMC9028831; doi:10.3390/plants11081088)
Supplement: Supplementary file 1 [file plants-11-01088-s001.zip › Supplementary Material 6.pptx]

## Slide 1
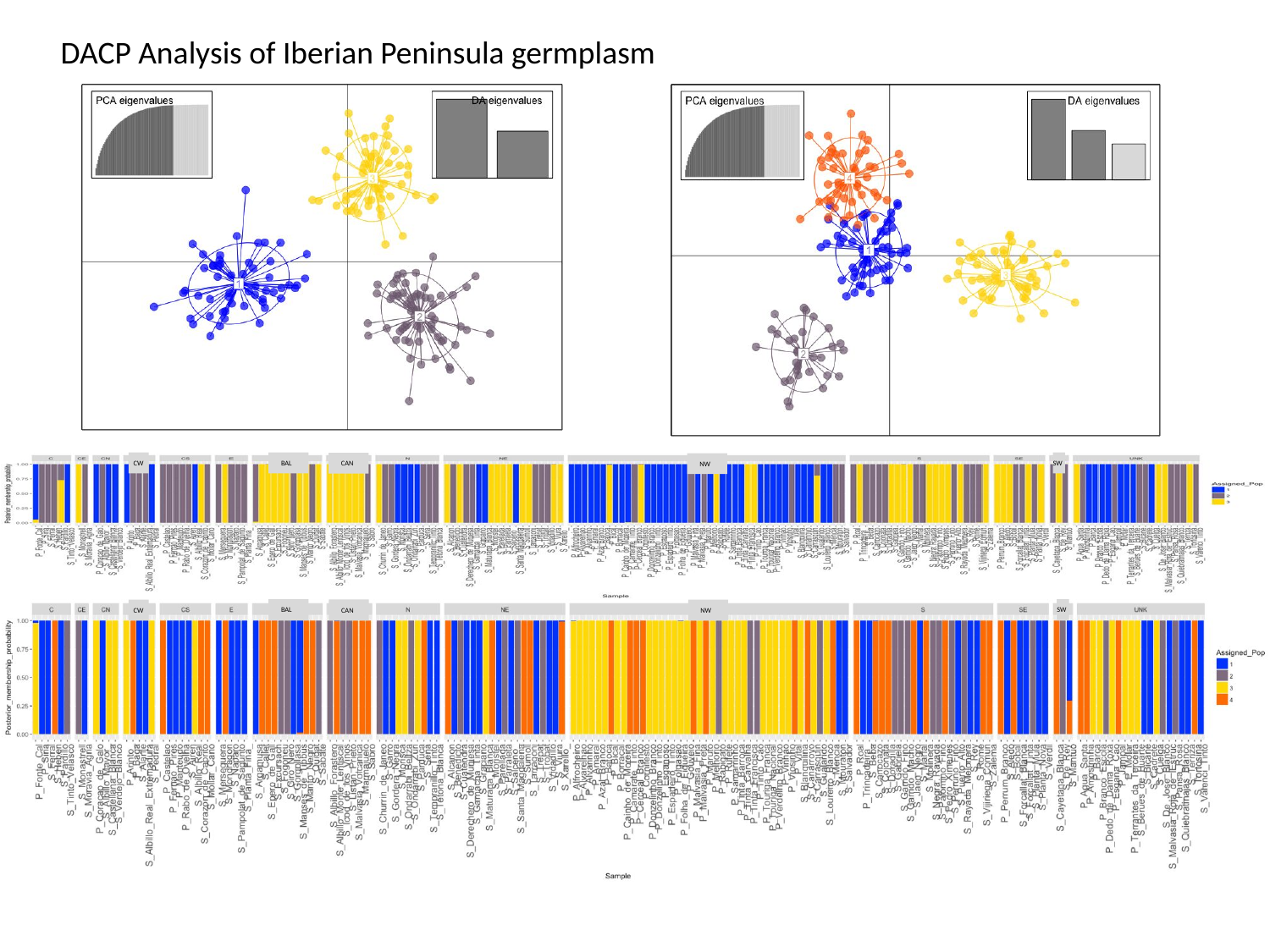

DACP Analysis of Iberian Peninsula germplasm
CW
BAL
SW
CAN
NW
SW
BAL
CAN
NW
CW

## Slide 2
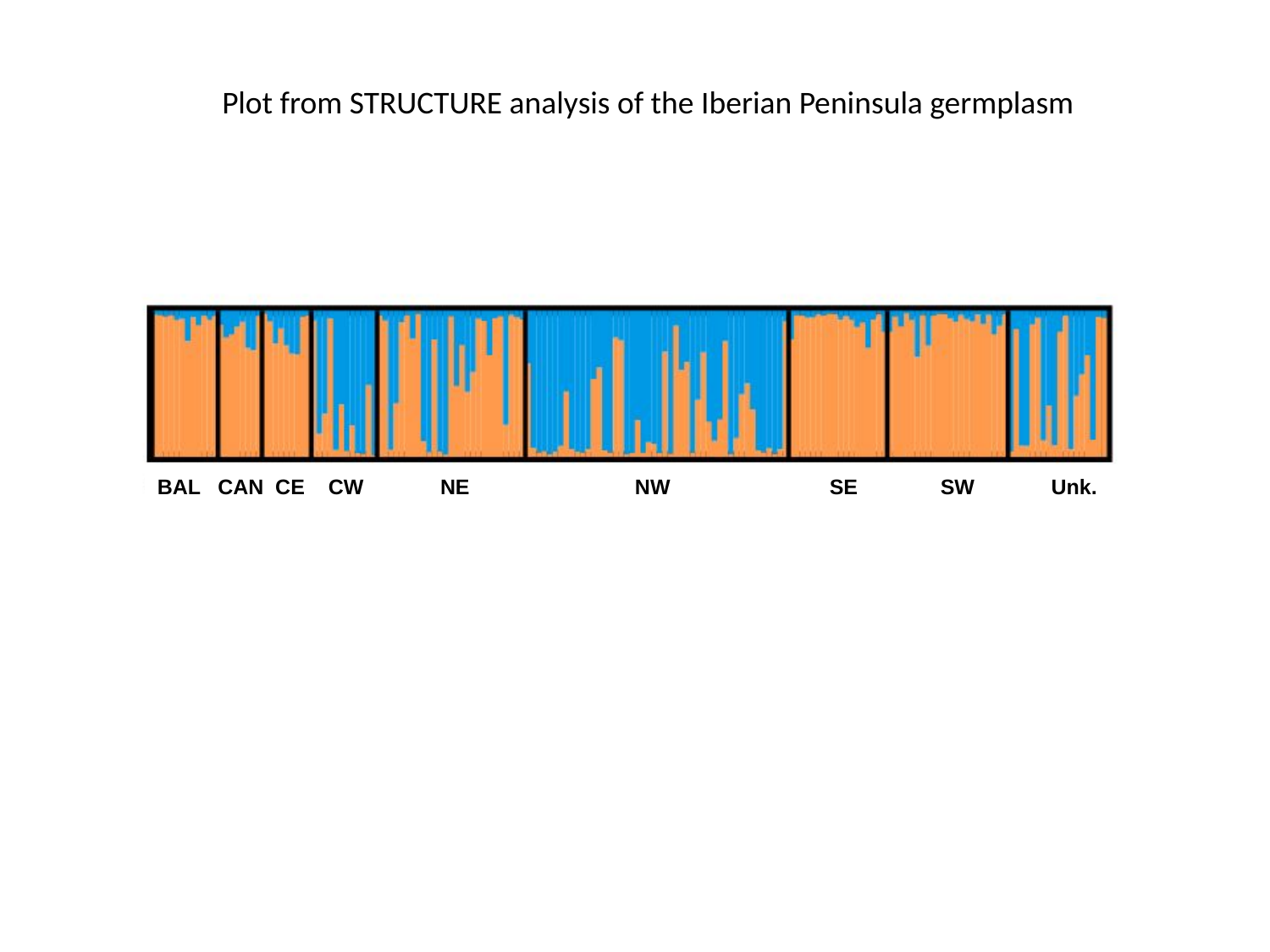

Plot from STRUCTURE analysis of the Iberian Peninsula germplasm
BAL CAN CE CW NE NW SE SW Unk.
