# Supplementary figures and images for: Genetic Characterization by SSR Markers of a Comprehensive Wine Grape Collection Conserved at Rancho de la Merced (Andalusia, Spain)
Source: Plants (Basel). 2022 Apr 16;11(8):1088. doi: 10.3390/plants11081088 (PMC9028831; doi:10.3390/plants11081088)

## Slide 1
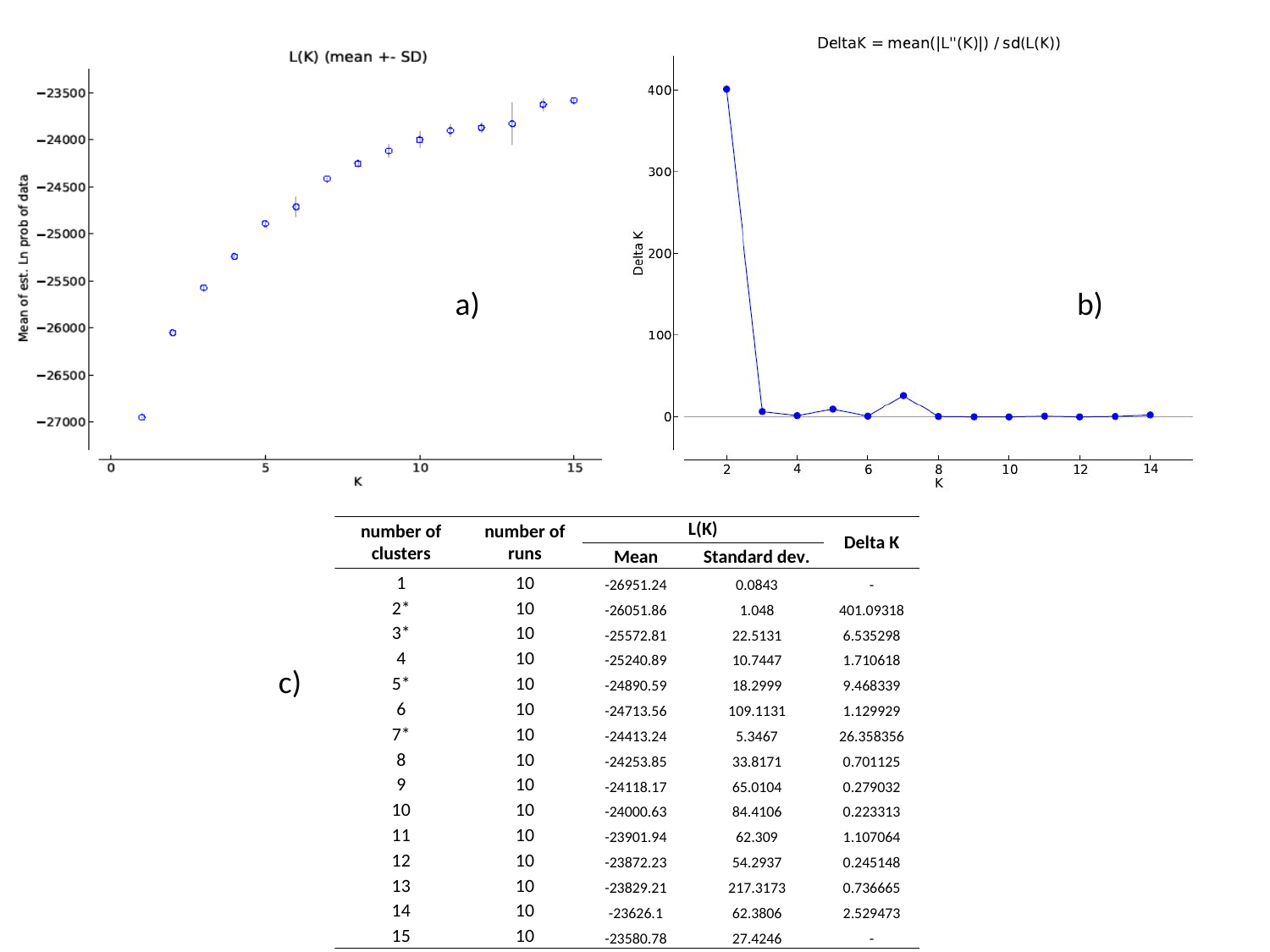

a)
b)
c)

Supplement: Supplementary file 1 [file plants-11-01088-s001.zip › Supplementary Material 2.pptx]

## Slide 1
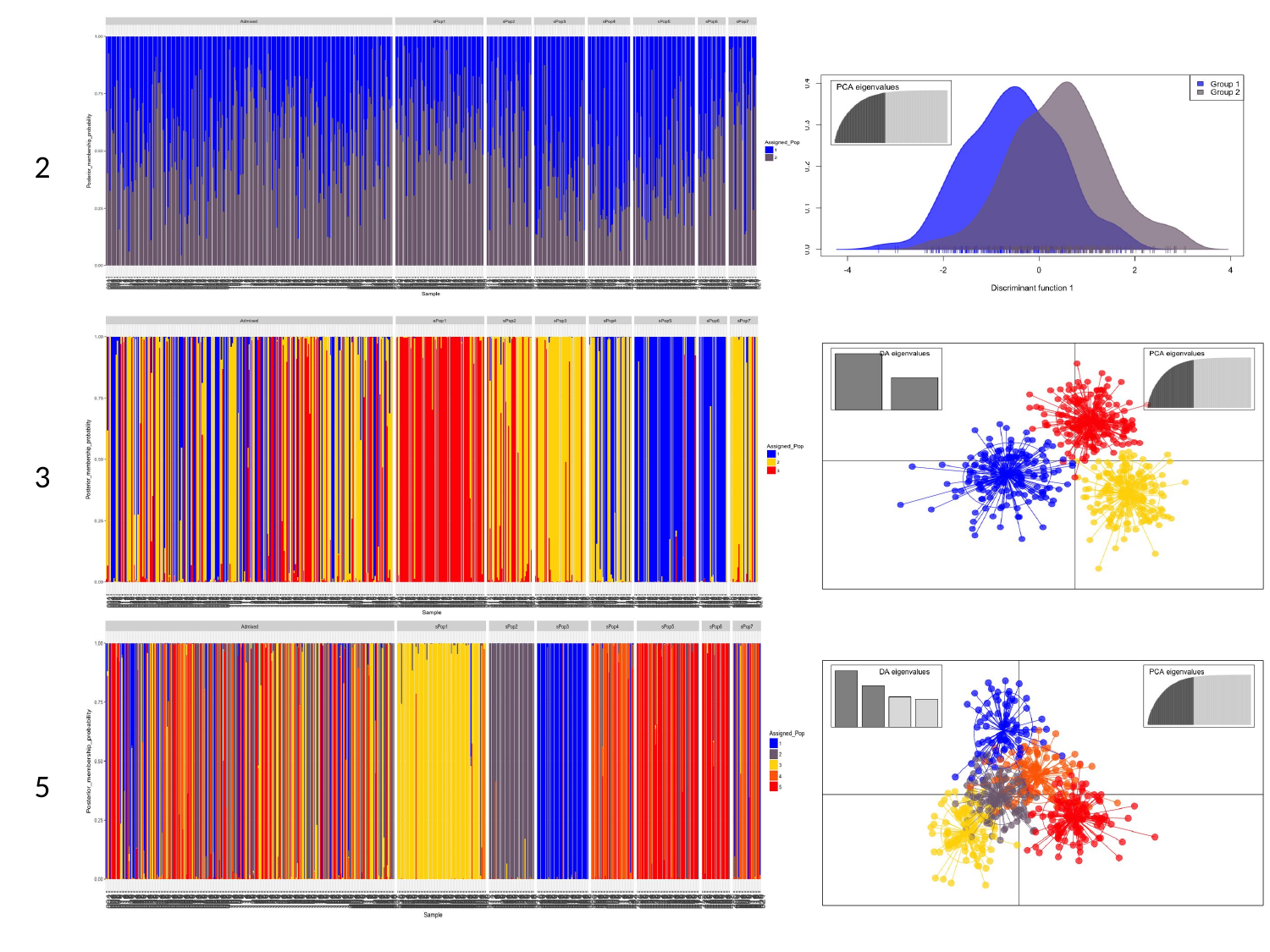

2
3
5

Supplement: Supplementary file 1 [file plants-11-01088-s001.zip › Supplementary Material 4.pptx]
